# Machine-learning prediction of biomass chemical composition using derivative thermogravimetric data of different biomass feedstocks

**DOI:** 10.1038/s41598-026-65442-3

**Published:** 2026-08-12

**Authors:** Satyajit Pattanayak, Dipankar Saha, Chanchal Loha, Kush Kumar Dewangan, Io Antonopoulou

**Affiliations:** 1 Biochemical Process Engineering, Division of Chemical Engineering, Department of Civil, Environmental and Natural Resources Engineering, Luleå University of Technology, 97187 Luleå, Sweden; 2 Department of Mechanical Engineering, School of Engineering, SR University, Warangal, 506371 India; 3 Energy Research Technology Group, CSIR-Central Mechanical Engineering Research Institute, Durgapur, West Bengal 713209 India; 4 Department of Mechanical Engineering, Indian Institute of Engineering Science and Technology, Shibpur, West Bengal 711103 India

**Keywords:** Biomass, Derivative thermogravimetry, Cellulose, Hemicellulose, Lignin, Machine learning, Cross-validation, Feature engineering, Energy science and technology, Engineering, Environmental sciences

## Abstract

The behaviour of biomass in bioenergy and biorefinery processes is determined by its contents of cellulose, hemicellulose, and lignin. The conventional wet chemical methods used to determine these fractions are reliable but time-consuming and laborious. This study attempts to determine whether these fractions can be adequately predicted from derivative thermogravimetric (DTG) data alone. A total of 75 biomass samples, including bamboo, agricultural residues, shells, and binary blends, were used. In addition, we calculated 13 simple, physically meaningful descriptors from each DTG curve (67 points from 27 to 687 °C), including peak height and temperature, areas under fixed-temperature windows, and area ratios. We trained seven algorithms, one for each component: Random Forest, Gradient Boosting, Extreme Gradient Boosting, Ridge, Partial Least Squares, Support Vector Regression, and k-Nearest Neighbours. All models were evaluated using nested leave-one-out cross-validation, with parameters optimised in the loop, and model stability was assessed with repeated fivefold cross-validation. The engineered descriptors improved every component. Cellulose was predicted with moderate accuracy (cross-validated R^2^ of about 0.50 to 0.56, RMSE about 6.8%). Hemicellulose was weaker (R^2^ about 0.38- 0.43, RMSE about 4.8%). Lignin could not be predicted reliably (stable R^2^ about 0.13). The reason is physical: lignin decomposes slowly over a wide temperature range that overlaps with those of the other two components. SHAP and permutation analysis tied the predictions to sensible temperature regions. The study shows what DTG-based prediction can and cannot do across feedstocks, and it provides an open, reproducible pipeline.

**Supplementary Information:**

The online version contains supplementary material available at 10.1038/s41598-026-65442-3.

## Introduction

The energy produced by burning biomass is a renewable energy source. It can be used to produce biofuels, bio-gas, and bio-based chemicals, which help to reduce greenhouse gas (GHG) emissions and dependence on fossil fuels^1,2^. There are many potential sources, such as forestry residues, energy crops, and agricultural by-products^1,3–7^. The same is true for every type of lignocellulosic feedstock: fast-growing species like bamboo are attractive, but their performance in a conversion process depends on their chemical composition.

Cellulose, hemicellulose, and lignin^8,9^ are the three major components of that composition. Fermentable sugars for bioethanol production are mainly derived from cellulose and hemicellulose. The breakdown of lignin is more difficult and can be converted to aromatic chemicals. Ideal pre-treatment conditions, higher biofuel yield, and better process economics are achieved by knowing the amounts of each^8–15^. They are normally measured by wet chemistry. It’s accurate, but slow, and consumes a lot of reagents. The spectroscopic techniques, such as FTIR or NIR, are faster but require calibration and do not transfer easily from one biomass type to another^11,16–23^. This has inspired researchers to attempt data-driven solutions^24^ to this problem.

Machine learning can capture the non-linear patterns in experimental data, and has proven effective in fields as varied as chemistry and toxicology^25^. It has been used for combustion prediction, gasification, hydrogen production, and biomass-property estimation^11,26–31^. Thermogravimetric analysis (TGA) records mass loss during heating, and its derivative (DTG) captures the decomposition of the three components in one fast measurement. Pairing DTG with machine learning is therefore a natural route to quick, non-destructive estimates of composition^8,24,31–35^. Interpretable methods such as SHAP can also show which temperature regions drive a prediction^36,37^. Ensemble tree methods (random forest, gradient boosting, and extreme gradient boosting) perform well on this type of data^24,32,33,36–43^. A brief survey of recent work is provided in the Supplementary Material (Table S4).

Some gaps remain. Several DTG and machine-learning studies are based on a single feedstock, a small dataset, and one train–test split. If the test is not truly out-of-sample, a single split could yield an overly positive result^1,44^. The R 2 values reported by different studies and components vary widely, and lignin is generally the most difficult to predict, as its decomposition is broad and overlaps with the others^45–48^. What is missing is work that is based on a variety of feedstocks, appropriately validates models with repeated cross-validation, and admits its own failures.

This study aims to address this gap. A DTG is made with 75 samples from various feedstocks. Physically meaningful descriptors are added to the raw DTG signal. We test seven algorithms, one algorithm per component, each with tuned settings, using nested cross-validation. We measure uncertainty using repeated fivefold cross-validation and interpret the models using SHAP and permutation importance. All conclusions are based on results from the out-of-sample data.

## Materials and methods

### Materials and sample collection

The 75 lignocellulose samples were selected from a variety of feedstocks to cover a wider range of compositions and thermal properties. A total of 75 samples were measured: 48 in our laboratory and 27 from the literature. The bamboo, rice straw, rice husk, mixed biomass, mixed leaf biomass, and binary blends of one biomass with another were all prepared and analyzed using the procedure described below. The remaining few samples were taken from published articles to extend the compositional range. The sources of these literature samples are listed in the Supplementary Material (Table S1). Details of the bamboo species, including the scientific names, are provided in the Supplementary Material (Table S1). No endangered or protected plant species were used, so CITES and IUCN guidelines did not apply.

The biomass samples were cut into small pieces and sun-dried for seven days. They were then dried in an electric oven at 353 K for 180 min to remove all moisture. Each experimental sample represented a distinct material, which preserved biological variation, and no replicate measurements of the same sample were included. The dried material was ground to a powder and sieved to a particle size of 90–120 μm; the fraction retained between the 120- and 140-mesh sieves is suitable for thermogravimetric analysis.

### Methodology

The work had four stages. First, we measured the reference cellulose, hemicellulose, and lignin contents by wet chemistry. Second, we recorded and processed the DTG signal. Third, we extracted features and built the machine-learning models with cross-validation. Fourth, we interpreted the models. Each stage is described below.

#### Determination of biomass composition

For all samples, we used wet chemistry to measure the structural composition, following the NREL Laboratory Analytical Procedure for structural carbohydrates and lignin^23,45^. So, the first step was to dry the biomass samples to a constant weight, add biomass fines, and then standardize sample size. The powder obtained was sieved to obtain the required particle size distribution, which was uniform for the experimental procedure. To obtain the soluble extractives, water was first used to extract them, followed by an ethanol–water solution to extract the structural carbohydrates, namely cellulose, hemicellulose, and lignin. The extracted biomass was then dried again to remove soluble compounds. Acid hydrolysis was carried out in two steps. The first process was conversion of 72% sulfuric acid at 30 °C for 1 h to hydrolyze hemicellulose mainly. Next, the concentration was reduced to 4% sulfuric acid, and the solution was autoclaved at 121 °C for 1 h. This step was followed by the hydrolysis of cellulose to glucose and other sugars in the liquid phase, yielding the hydrolysate. During the hydrolysis mentioned above, lignin, which was insoluble in acid hydrolysis, remained as a residue. This solid fraction is known as the acid-insoluble lignin. After hydrolysis, the lignin was floated from the liquid hydrolysate, and the monosaccharides were derived from cellulose and hemicellulose. The sample was washed and filtered to remove any remaining acid; the lignin mass was recorded until the mass became constant. The above total biomass weight of this dried residue was used to quantify the proportion of lignin in the raw biomaterial examined. The structural composition analysis results for all bamboo samples are provided in the supplementary material table S1.

#### Thermogravimetric analysis and DTG data

The biomass samples were sundried for 7 days after cutting them into small pieces. The water content in the samples was completely removed by drying for 180 min at 353 K in an electric oven. Biological variation was accounted for by treating each sample as representing one plant. There were no remeasurements of the same sample. The dried bamboo samples were ground into powder and then sieved to obtain particles with a diameter range of 90–120 μm (between 140- and 120-mesh sieves), suitable for TGA. The thermal gravimetric analysis (TGA) was carried out with the NETZSCH TG 209 F3 Tarsus TGA apparatus. Fine bamboo powder was placed in the small alumina crucible, and non-isothermal heating was carried out at different heating rates (10, 20, 30, and 40 K/min) from room temperature to 700 °C. Nitrogen was set at 40 mL/min to ensure all reactions were done in an inert environment. As a result, there was no oxidation. Data on the sample’s weight loss were obtained during heating (thermogravimetric analysis, TGA) and yielded derivative thermogravimetric (DTG) data. All the experiments were carried out at least three times to ensure the accuracy and reproducibility of the results. DTG data were collected for this study at 10 °C intervals from room temperature to 700 °C at a heating rate of 10 K/min. All the DTG data for the bamboo samples are provided in the supplementary material; the selected DTG temperature data points were 67 out of 671.

#### Feature extraction and engineering

We compared two feature sets. The raw set was the 67 DTG values. The augmented set added 13 descriptors, each computed from one sample’s own curve so that no information is shared between samples. These were: the maximum mass-loss rate and its temperature; the mean and standard deviation of the DTG signal; the rate and integrated area in three windows (150 to 275 °C, 275 to 400 °C and 400 to 600 °C, for the hemicellulose, cellulose and lignin/char regions^42–44^); the cellulose-to-hemicellulose and lignin-to-cellulose area ratios; and the width of the main peak. The area in a window is roughly proportional to the mass lost there, so these descriptors point more directly at how much of each component is present than any single raw point does.

#### Machine-learning algorithms

Seven algorithms in various families have been tested. Random forest (RF) is an average of many bootstrapped decision trees that helps to minimize variance and is relatively resistant to outliers. Gradient boosting (GB) and extreme gradient boosting (XGB) are sequential tree constructions corrected for errors, with XGB introducing regularisation^45^. Ridge regression is a linear model with an L2 penalty, useful when predictors are correlated. Partial least squares (PLS) projects the inputs onto a few latent variables and is common in chemometrics. Support-vector regression (SVR) fits a kernel-based margin. K-nearest neighbors (kNN) predict from nearby samples. Together, these cover linear, latent-variable, kernel, instance-based, and ensemble approaches.

#### Single-target modeling strategy

We trained a separate model for each target. This lets the algorithm and its settings be tuned for cellulose, hemicellulose, and lignin individually. The alternative, one model predicting all three at once, ties them together. Because the three components differ greatly in how well they can be predicted, separate models yielded better results and a clearer picture of each.

#### Model training, hyperparameter optimization and cross-validation

Features were standardized to zero mean and unit variance using only the training data, so no test information leaked in. We tuned the settings with nested cross-validation. The outer loop was leave-one-out, which yields an unbiased out-of-sample error estimate. The inner loop was a fivefold grid search that picked the settings using only the outer training data. We then ran repeated fivefold cross-validation (10 repeats) and report the mean and standard deviation of the fold scores. In k-fold cross-validation, the data are split into k parts; the model trains on k − 1 parts and is tested on the one left out, rotating through all k folds until every sample is tested once. Leave-one-out is the case where k equals the number of samples. On a small dataset, reporting the spread of repeated folds, not just one split, is what separates a real result from a lucky one.

The inner grid search tuned the main hyperparameters of each algorithm. Random forest used 300 trees, with maximum depth set to 3, 4, or unlimited, and minimum leaf size to 1 or 2. Gradient boosting used 200 trees, with depths of 2 or 3 and learning rates of 0.03, 0.05, or 0.1. Extreme gradient boosting used 200 trees, with depth set to 2 or 3, the learning rate to 0.05 or 0.1, and the L2 penalty to 1 or 3. Ridge chose its L2 strength from thirty values spaced logarithmically between 10^−2^ and 10^4^. Partial least squares chose among 3, 5, 8, or 12 latent components. Support-vector regression chose the penalty C from 1, 10, or 50 and the kernel width from the scale heuristic or 0.01. K-nearest neighbors chose the number of neighbors from 3, 5, or 7. Within each outer training fold, the selected setting was the one with the lowest mean squared error in the inner fivefold search. For the reported models, this gave a random forest with 300 trees and depth 4 for cellulose, five neighbors for hemicellulose, and support-vector regression with C = 10 and the scale kernel width for lignin. The full grids and the values selected for every model are given in the deposited code.

#### Performance metrics

We report three metrics, all from held-out predictions and on the original scale (%): the coefficient of determination (R^2^), the root-mean-square error (RMSE), and the mean absolute error (MAE):

1 $${R}^{2}=1-\frac{\sum_{i=1}^{N}{\left({y}_{i}^{exp}-{y}_{i}^{pred}\right)}^{2}}{\sum_{i=1}^{N}{\left({y}_{i}^{exp}-{\overline{y} }_{i}^{exp}\right)}^{2}}$$

2 $$RMSE=\sqrt{\frac{\sum_{i=1}^{N}{\left({y}_{i}^{exp}-{y}_{i}^{pred}\right)}^{2}}{N}}$$

3 $$MAE=\frac{\sum_{i=1}^{N}\left|{y}_{i}^{exp}-{y}_{i}^{pred}\right|}{N}$$

Here, ${y}_{i}^{exp}$ and ${y}_{i}^{pred}$ are the measured and predicted values, ${\overline{y} }_{i}^{exp}$ is the mean of the measured values, and *N* is the number of samples. We report RMSE as the square root of the mean squared error, so it has the same units as the target and is always at least as large as the MAE.

#### Feature-importance analysis

We measured how much each feature mattered using SHAP values for the tree models and permutation importance for the others, applied to the best model for each target. SHAP gives each feature its average contribution across feature subsets^40^. We ranked features by their mean absolute SHAP value. A positive value increases the predicted composition, and a negative value decreases it.

#### Software and code availability

All analysis was done in Python with scikit-learn and XGBoost. All steps of the pipeline: feature extraction, cross-validation, uncertainty estimation, and figure generation are provided at 10.5281/zenodo.21130334.

## Results and discussion

### DTG behavior and its relation to composition

**Fig. 1:**
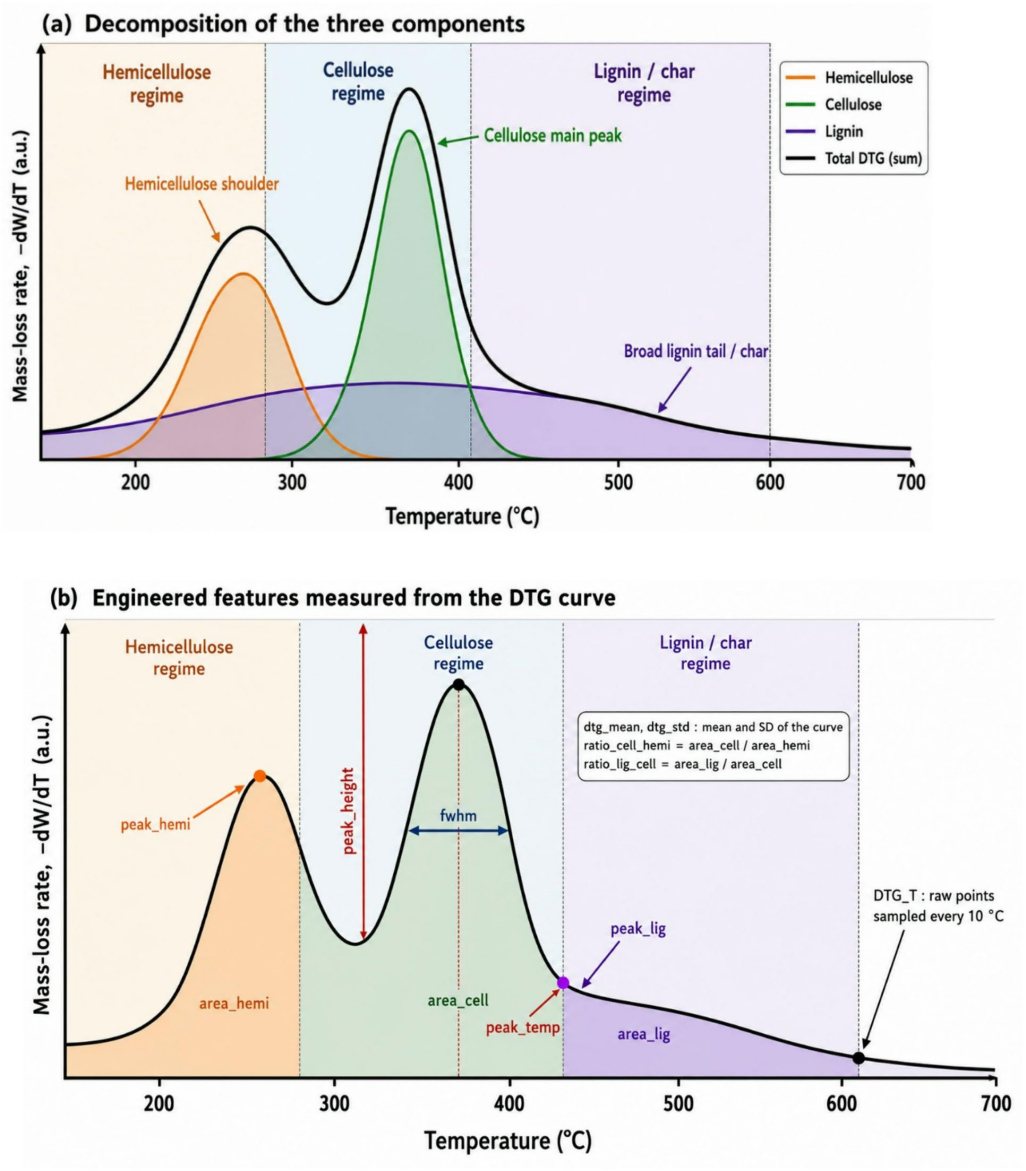
Decomposition of biomass and feature definitions. (**a**) The schematic shows the contributions of hemicellulose (shaded), cellulose (shaded), and lignin (shaded), as well as the sum of these contributions (the total DTG curve, black), with the three temperature regimes marked. (**b**) The engineered features measured from the DTG curve: the peak height and temperature (peak_height, peak_temp); the full width at half maximum (fwhm); the integrated areas and within-window peaks for the hemicellulose, cellulose and lignin windows (area_hemi/area_cell/area_lig and peak_hemi/peak_cell/peak_lig); the raw points sampled every 10 °C (DTG_T); and the derived ratios and curve statistics. The curves in (a) are not actual measurements, but rather typical curves of a component.

The DTG curve shows the mass loss as a function of temperature and is divided into three regions. This is shown in Fig. 1, which presents a schematic of the three component contributions and their sum. Hemicellulose is the least refractory and breaks down first, at 200–315 °C, and is often present as a shoulder on the primary peak^46,47^. Cellulose is also crystalline and has a narrower, higher decomposition temperature range, 315 to 400 °C, with the main peak^47^. Lignin is an aromatic polymer with cross-links. Rather than a sharp peak, it decomposes slowly from below 200 °C to above 600 °C, producing a broad, low-intensity peak at the end of the mass spectrum as char. It does not have a sharp peak; it decomposes gradually from below 200 °C to above 600 °C, generating a broad, low-intensity peak at the end of the mass spectrum as char.

The figure says it all. The measured DTG curve results from the three overlapping contributions. The amount of cellulose is the easiest to read from the curve, as it produces the tallest, sharpest, and best-separated peak. The hemicellulose shoulder lies on the rising side of the cellulose peak, making the two difficult to separate. Lignin is not concentrated in one location and does not produce a distinct peak in the signal, so it appears as a general background. This is the reason why the three components are not equally predictable.

The sequence cellulose first, hemicellulose, and then lignin follows directly from the nature of the clear signature that each leaves. This ordering is the reason the following predictions are made and accord with previous TGA-derived compositional studies^8,47,48^. The three shaded regions in Fig. 1 are also the temperature window used to construct area and peak features (Sect. "Feature extraction and engineering"). Figure 1b shows the locations of these features on the curve, and they are represented on the axes of the importance plots in Sect. "Feature-importance interpretation".

### Effect of engineered features

**Fig. 2:**
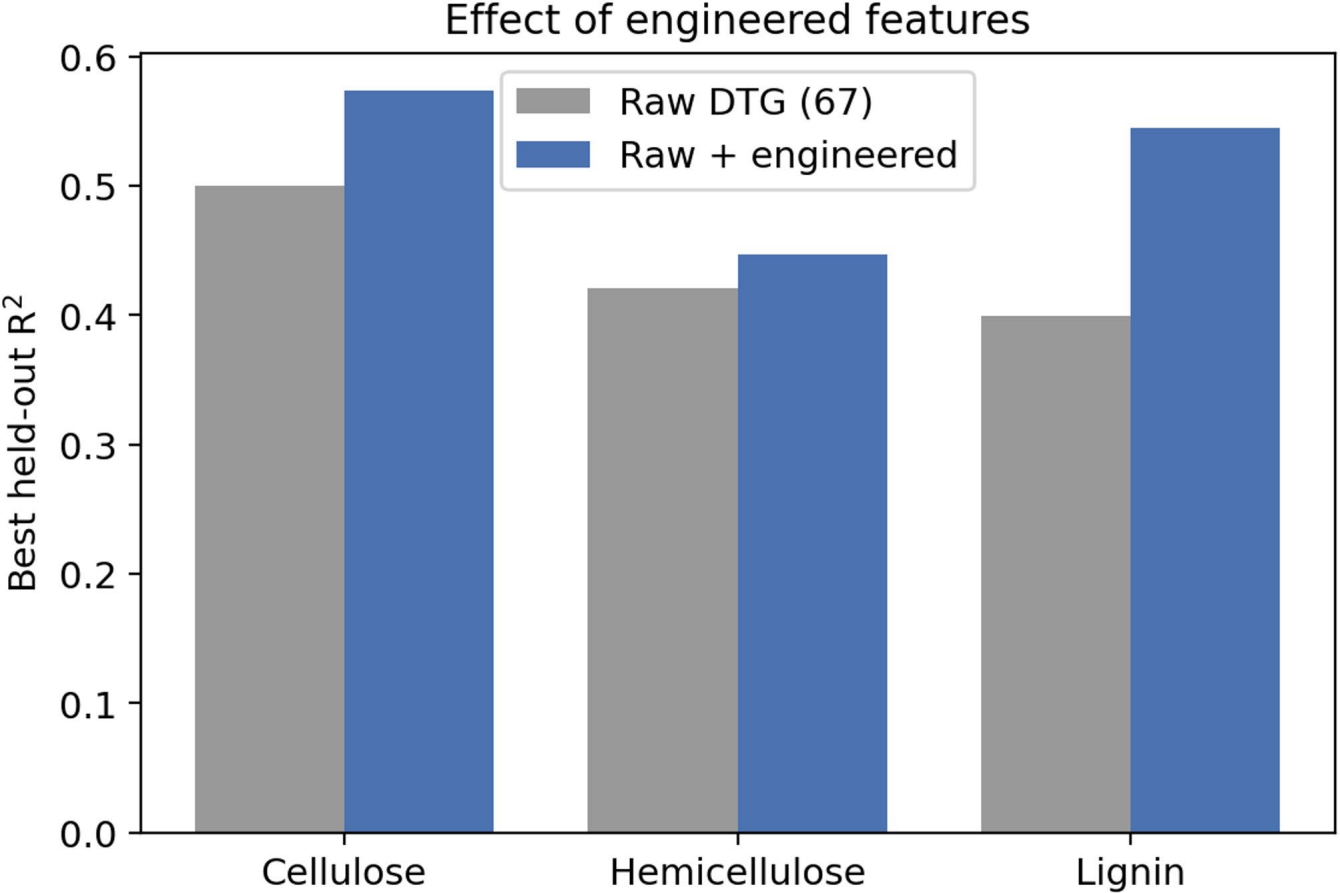
Held-out R^2^ for the reported model of each component, using raw DTG features (67 points) versus raw features plus engineered descriptors.

The 13 descriptors were added to the raw DTG points, which enhanced all components (Fig. 2, Table 1). The leave-one-out R^2^ for each component was increased from 0.44 to 0.56 for cellulose, from 0.33 to 0.43 for hemicellulose, and from 0.19 to 0.22 for lignin. It’s simply because of this. The window-area and peak descriptors integrate the component’s mass loss over its range, thus providing a better indication of the amount of component present than a single raw data point alone. This is consistent with regular chemometric experience that a small number of good descriptors is as effective as, or more effective than, a collection of many raw data inputs^8,44,47^.

**Table 1:** Effect of engineered features on held-out R^2^ (leave-one-out CV) for the reported model of each component.

Component	Raw DTG (67)	Raw + engineered
Cellulose	0.44	0.56
Hemicellulose	0.33	0.43
Lignin	0.19	0.22

The size of the gain depends on the clarity of each signal. The sharp event between 315 and 400 °C in the thermogram is captured directly by the cellulose window area, and so the cellulose also gained the most from 0.44 to 0.56. The decline in hemicellulose (0.33–0.43) is low due to the overlap of its decomposition onset with that of cellulose, and there is no window in which its decomposition can be isolated. There was only slight movement for lignin, from 0.19 to 0.22, since there is no clear peak to target a descriptor.

The absolute R^2^ values are moderate: it should be worthwhile to determine the reason. The first reason is: DTG does not measure composition directly. This measures the rate of mass loss, a kinetic indicator. The connection between this signal and the quantity of each polymer is indirect, and several effects attenuate it. The three biopolymers do not break down individually. They interact with each other and with the inorganic minerals in the biomass during pyrolysis, and the inorganic minerals catalyze and drive the pyrolysis reactions. The measured curve will therefore not be a “pure” linear combination of three established, fixed component curves^46–48^. For this reason, within a temperature window, the area is only approximately equal to the matching component. This approximation can be used to define the upper bound for the performance of any model constructed from the features.

The second is the dispersion of the data set. This consists of 75 samples from various feedstocks and blends, measured at a variety of heating rates (10- 40 K min^−1^). The DTG peaks shift to higher temperatures and become broader due to thermal lag; higher heating rates and different feedstocks would yield different ash and mineral contents, shifting the DTG peaks accordingly. This is intentional, since it makes the models general, but there is also some variation between two samples with similar composition that produce somewhat different curves. This additional scattering reduces the value of R^2^ compared with a study of a single feedstock at a single heating rate, which shows a closer relationship between the curve and composition but does not necessarily generalize to other materials^1,44^.

The third reason is the noise in the reference values. Cellulose, hemicellulose, and lignin contents were determined by wet-chemical analysis with reproducibility of a few percent in absolute values, and a few samples were obtained from other laboratories and analyzed by other methods. A better model cannot predict a target more accurately than the target is known. Not all of the unexplained variance is due to the model’s failure; some is due to measurement noise in the labels. The acid-insoluble residue is the least clean of the three. It has the highest label-noise ceiling, as both the signal and reference value may contain ash and condensed pyrolysis products. In this context, a cross-validated cellulose R^2^ of ~ 0.5 is a good and honest result, and the engineered features enhanced all components; therefore, we retained them for further analysis.

### Model comparison and per-component performance

**Fig. 3:**
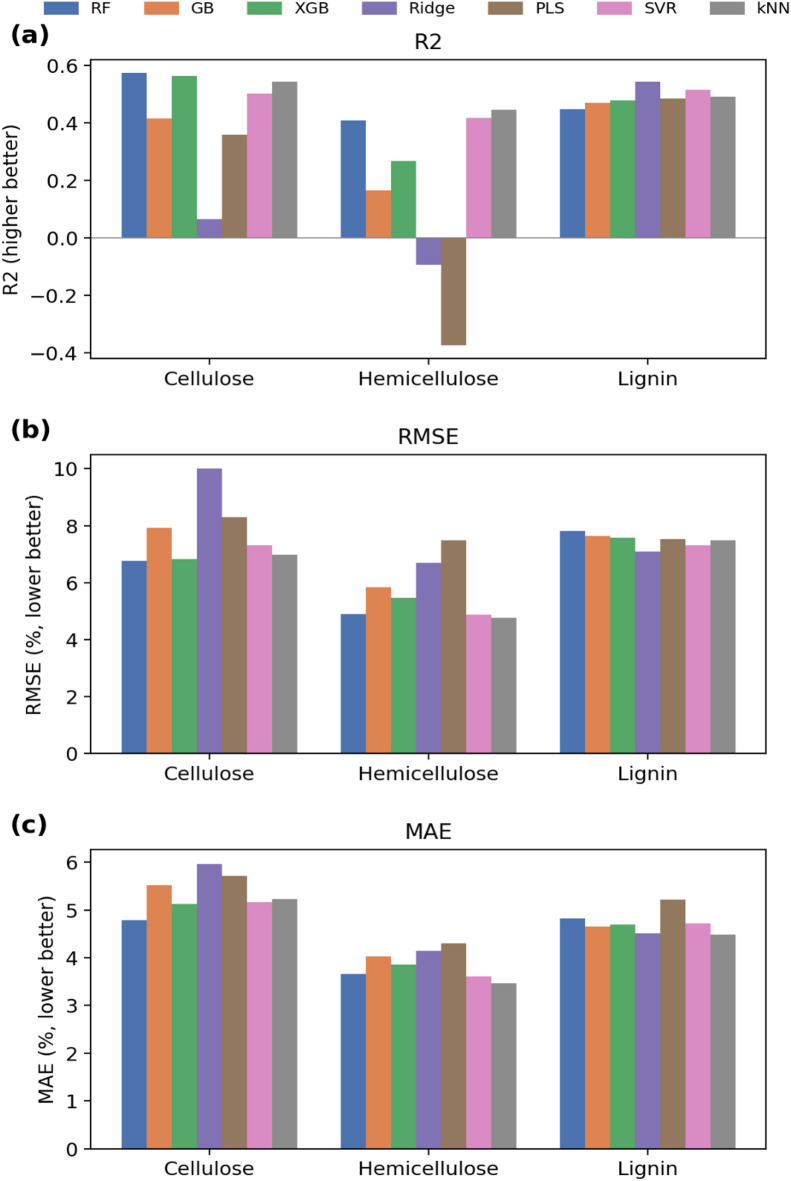
Model comparison for the engineered feature set on a common scale: (**a**) R^2^, (**b**) RMSE and (**c**) MAE for each component across the seven algorithms.

Figure 3 compares the seven algorithms, and Table 2 presents the best model for each component along with the cross-validation scores. The algorithms are only in a relatively small range for each component. This is a statement about the limit, not about the selection of the model, but about the information in the DTG signal. This is the same trend seen in other biomass studies with good features^24,32^. The best model for cellulose was random forest, hemicellulose was best with kNN, and the most stable lignin estimate was obtained with SVR. The ability of RF and SVR to perform well is consistent with the literature: RF is found to work well on small, noisy datasets^49^, and kernel and boosting methods are effective for non-linear signals^24,50^. Ridge, a linear model, performed poorly on the hemicellulose data, indicating that the relationship is nonlinear. No single algorithm won on all three targets. This is expected: by the no-free-lunch theorem, no learner is optimal for every problem, and the best choice depends on the data’s structure^51^. We therefore used a separate model for each component.

**Table 2:** Best model and held-out performance per component (engineered features, 75 samples).

Component	Best model	LOO-CV R^2^	Repeated fivefold R^2^ (mean ± SD)	RMSE (%)
Cellulose	RF	0.56	0.50 ± 0.22	6.8
Hemicellulose	kNN	0.43	0.38 ± 0.22	4.8
Lignin	SVR	0.22	0.13 ± 0.23	7.3

The repeated fivefold result is the primary, robustness-checked metric. A ridge model reached a higher single leave-one-out R^2^ for lignin (0.55) but was unstable under repeated cross-validation (− 0.24 ± 1.63), so the stable SVR estimate is reported.

The panels in Fig. 3 agree with this. In the R^2^ panel (Fig. 3a), RF, XGB, SVR, and kNN form a tight high group for cellulose, while ridge and PLS trail. For hemicellulose, the linear models drop below zero, which again shows a linear fit is not enough—the RMSE and MAE panels (Fig. 3b, c) match. Cellulose has the largest errors because it spans the widest range (about 6–64%). Hemicellulose has the smallest RMSE (about 4.8%) because its range is narrow. Lignin has a high RMSE and a low R^2^ at the same time, which is what a model does when it is little better than guessing the mean. Table 2 also puts the two validation scores side by side. For cellulose and hemicellulose, the values agree (0.56 vs 0.50 and 0.43 vs 0.38), indicating stable results. For lignin, they disagree, which Sect. "Robustness and the difficulty of lignin" explains.

The negative values for some models warrant a word, as they seem surprising. R^2^ measures a model against the simplest baseline, predicting the mean of the data every time. R^2^ = 0 means the model performs no better than the baseline, and R^2^ < 0 means it performs worse^52^. A negative value is therefore not a small error. It signals that the model has found no usable relationship and is misfitting the held-out samples. Two things drive it here. First, ridge and PLS are linear models, and the hemicellulose-to-DTG relationship is not linear, because the hemicellulose signal overlaps the cellulose onset. Forced through many correlated inputs, a linear model fits training noise and then predicts worse than the mean out-of-sample, so its score drops below zero. Second, the leave-one-out scheme itself is ill-suited to strongly regularised models. When one sample is held out, the mean of the remaining training labels shifts slightly away from that sample, and models that pull their predictions toward the training mean, as a heavily regularised linear model does, are penalized for it. This distributional bias has been shown to lower, and even invert, cross-validated scores, and to act most strongly on the most regularised models^52^. It explains why ridge and PLS fall below zero for hemicellulose, and the same effect underlies the unstable lignin result in Sect. "Robustness and the difficulty of lignin". Reporting repeated k-fold scores alongside leave-one-out, as we do, is the safeguard against being misled by it^52^.

### Prediction quality

**Fig. 4:**
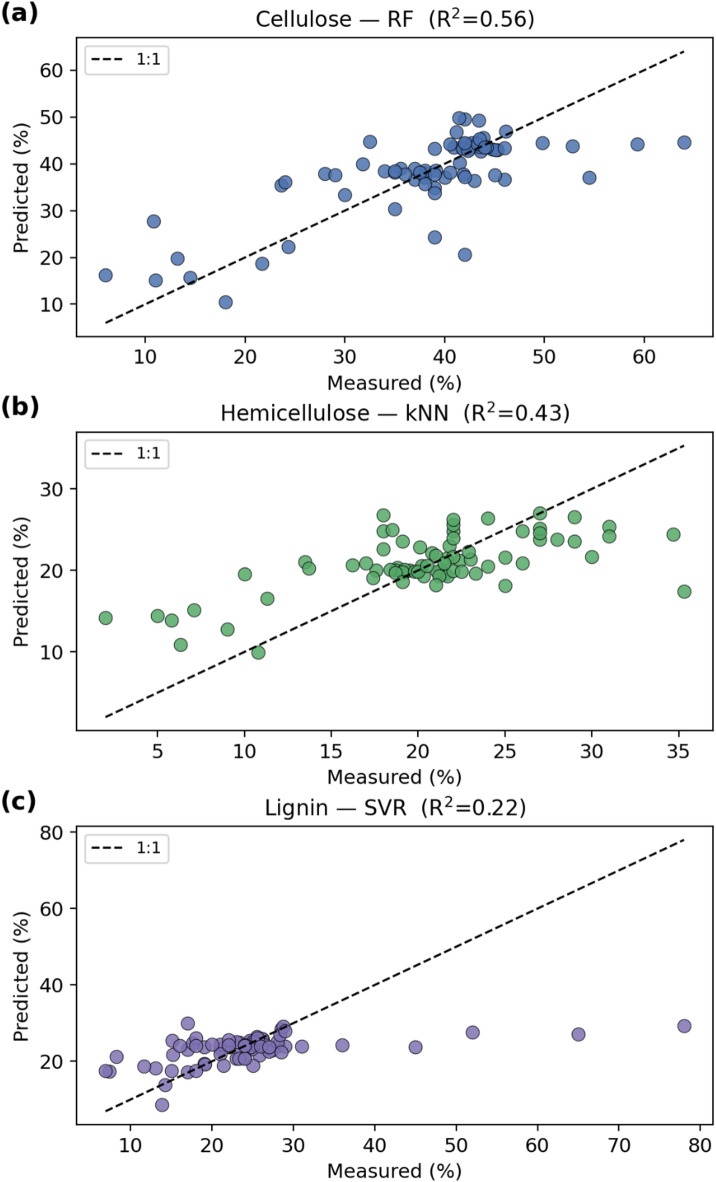
Held-out predicted versus measured composition (nested leave-one-out CV): (**a**) cellulose, (**b**) hemicellulose, (**c**) lignin. Dashed line: 1:1

**Fig. 5:**
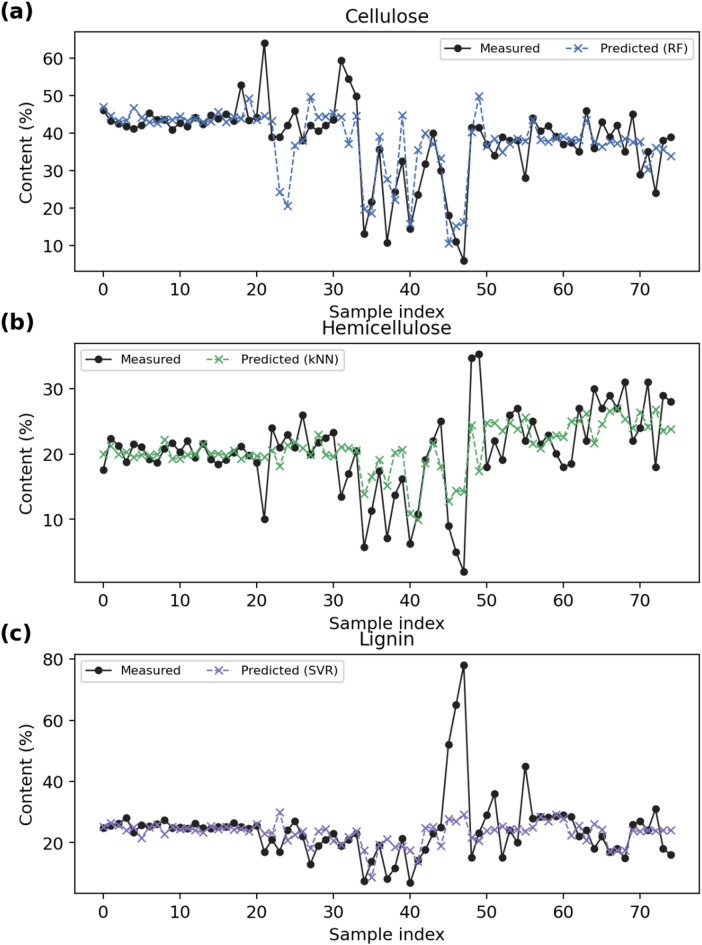
Measured (solid) and held-out predicted (dashed) composition across all samples: (**a**) cellulose, (**b**) hemicellulose, (**c**) lignin.

**Fig. 6:**
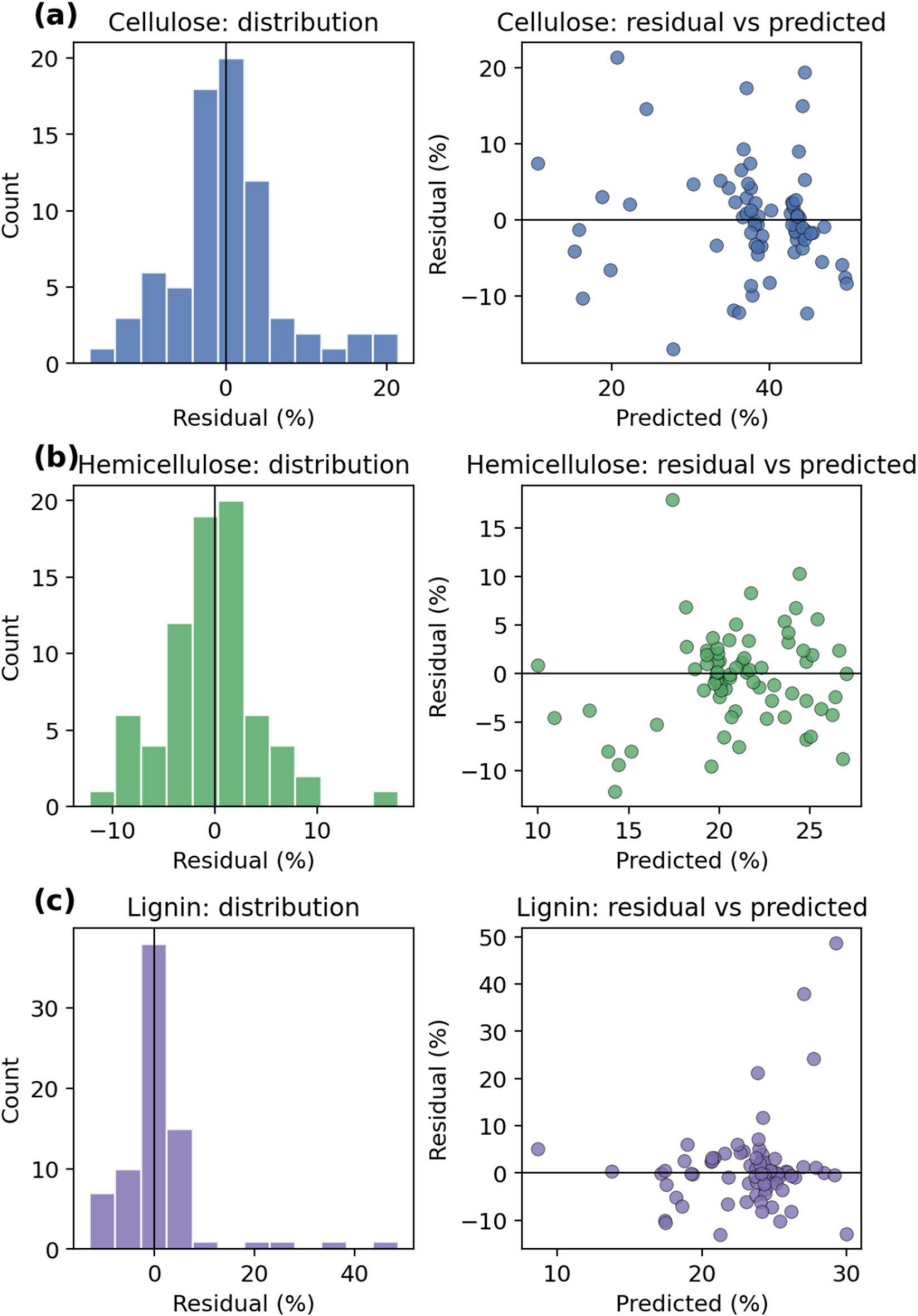
Residual distributions (left) and residuals versus predicted values (right) for (**a**) cellulose, (**b**) hemicellulose and (**c**) lignin.

We assessed prediction quality in three ways: the predicted-versus-measured plot (Fig. 4), the sample-by-sample trace (Fig. 5), and the residuals (Fig. 6).

The parity plots are shown in Fig. 4. In the case of cellulose (Fig. 4a), the points are distributed along the 1:1 line in the whole range (around 6–64%). There is greater scatter at the extremes, where both the blends and the char-rich samples are found, and the trend is the same, with R^2^ values of 0.50–0.56. The model is monitoring actual changes in cellulose, rather than predicting an average value. In the case of hemicellulose (Fig. 4b), the cloud is looser, but still follows the diagonal, and has a lower R^2^ of around 0.38–0.43. In the case of lignin (Fig. 4c), all points are grouped within a very narrow range around the dataset mean. This averaging down is to be predicted when the features contain little information on the target. Thus, under squared-error loss, the safest prediction is to shrink to the average, and a weak-signal target such as lignin will be drawn to the center^52^. The model performs well for the central cluster (15–30%) but cannot track the high-lignin char samples. The samples are located at the very sparse end of the data set, beyond the model’s applicability domain, and thus extrapolation outside that range is unreliable^53^.

The measured and predicted values are plotted in Fig. 5, one at a time, in turn. The cellulose plot (Fig. 5a) shows reasonable agreement between the predicted and measured values, including the sharp decreases observed in the blends (samples 34 to 47). The model reflects big swings, not averages. In the case of hemicellulose (Fig. 5b), the general trend is captured, but the highest and lowest values are smoothed. In the case of lignin (Fig. 5c), the fitted line is nearly flat and does not capture the large upward-measured peaks present in the char samples (e.g., the peak near sample 47). This is the same failure observed in the parity plot. Again, the flat lignin trace is the signature of regression to the mean; the tall char spikes are high-leverage points that are sufficiently removed from the bulk of the data and are just two peaks apart, allowing them to be reached by the model^52,53^.

Figure 6 shows the residuals. For cellulose and hemicellulose (Fig. 6a, b), the histograms are roughly symmetric and centered on zero, and the residual-versus-predicted plots show no clear trend. So, these two models have little systematic bias and fairly even, or homoscedastic, errors, which is what a well-specified model should show. The lignin residues (Fig. 6c) differ. They have heavy tails, with several large positive values from the under-predicted char samples, and the spread grows with the predicted value (heteroscedasticity). The positive skew means the model under-predicts the high-lignin samples in a systematic way rather than missing at random. Both features point to the same cause. The high-lignin region is sparsely sampled, and the lignin signal in the DTG curve is weak and overlapping^46–48^, so the model is mis-specified exactly where the values are largest and of most interest^54^.

### Robustness and the difficulty of lignin

**Fig. 7:**
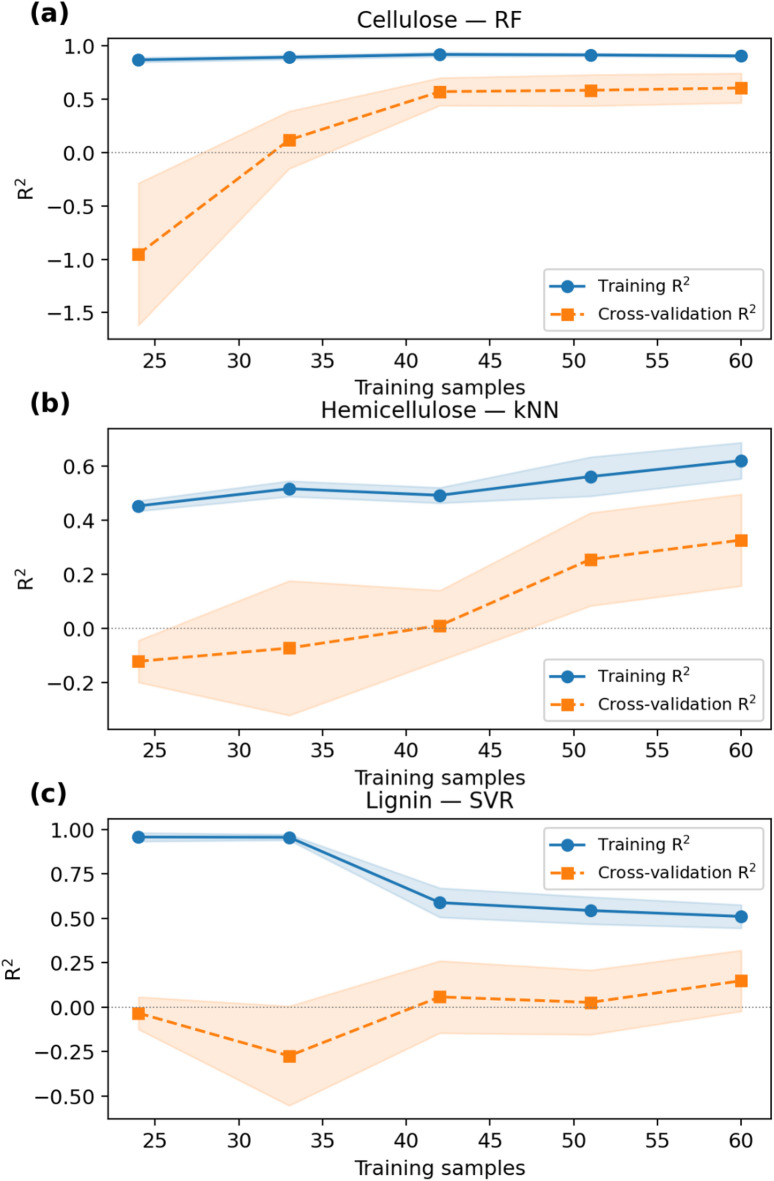
Learning curves (training vs. cross-validation R^2^) for the best model of each component: (**a**) cellulose, (**b**) hemicellulose, (**c**) lignin.

The learning curves (Fig. 7) show the gap between training and cross-validation scores closing as more data is added. A closing gap shows the models are not badly over-fitted at this sample size, and the bias-variance behavior of the curves shows where more data would help most^55^. The key point is about lignin. Its two validation scores disagree sharply. A ridge model reached a leave-one-out R^2^ of 0.55, but the same model gave a repeated fivefold R^2^ of − 0.24 ± 1.63. The mean is below zero, and the spread is larger than the mean, so that 0.55 was not reproducible. It came from leave-one-out being too easy when a single high-lignin char sample is held out at a time. SVR gave the only stable lignin score (about 0.13 ± 0.23), so we report that. The disagreement is statistical rather than an error. Leave-one-out has low bias but high variance as an estimator of generalization error, and pooling the score over folds that each hold out a single point makes it sensitive to a few influential samples^52^. Using the same cross-validation to both tune and score a model also yields optimistic results, which is why nested cross-validation, as used here, and repeated k-fold give more honest, lower-variance estimates^54^. Under repeated fivefold, the high-lignin char samples end up in test folds the model cannot reach, and a few such folds pull the average down, which is reflected in the large standard deviation. The single leave-one-out value of 0.55 was therefore an artifact of the estimator, not real predictive skill. This is the clearest example in the study of why repeated validation matters on small data. One split, or one leave-one-out pass, can show a decent R^2^ for a target that cannot really be predicted. The difficulty of lignin is also well known: its broad, overlapping decomposition leaves little lignin-specific information in the DTG signal^46–48^, and earlier studies report lignin as the hardest fraction, with much lower R^2^ than cellulose^13,44^.

Each curve tells its own story. For cellulose (Fig. 7a), the cross-validation score rises steadily with more data and the gap to the training score narrows. A closing gap like this is the signature of a variance-limited model that is still learning, so more data would raise its score further^56^. For hemicellulose (Fig. 7b), the score also rises but levels off at a lower value. A plateau like this indicates bias rather than variance: the model has captured about as much of the DTG signal as that component contains, so adding similar samples yields little further gain^55^. For lignin (Fig. 7c), the score remains low, fluctuates, and does not converge on the training score, so more data of the same kind is unlikely to fix it. In short, more samples would help cellulose and, to a lesser extent, hemicellulose, but lignin would need a different measurement as well.

### Feature-importance interpretation

**Fig. 8:**
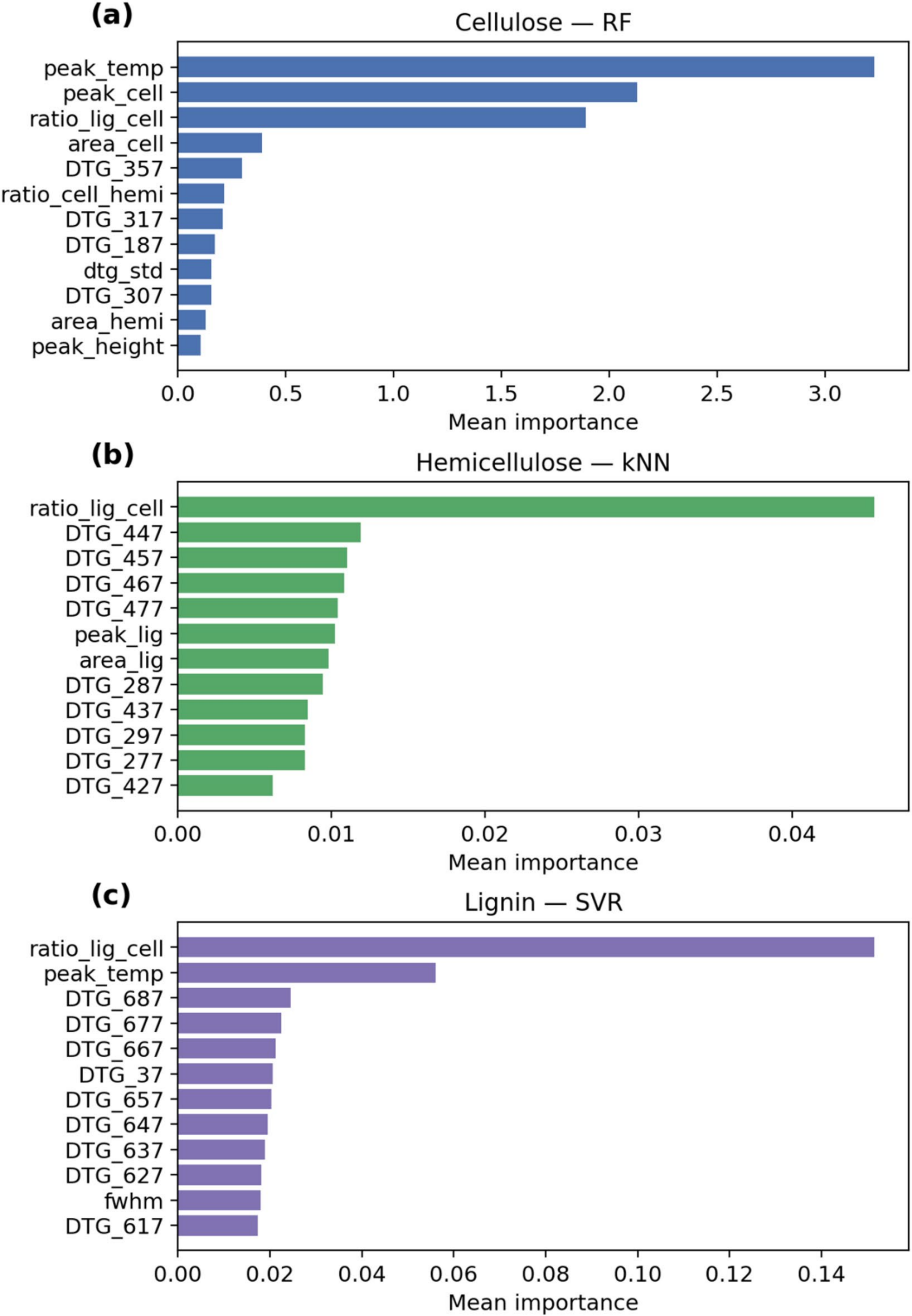
Feature importance (SHAP for tree models; permutation importance otherwise) for the best model of each component: (**a**) cellulose, (**b**) hemicellulose, (**c**) lignin.

Figure 8 links the predictions to specific features. The engineered descriptors on the vertical axes are defined as follows, and Fig. 1b shows where each is measured on the DTG curve. peak_height is the maximum mass-loss rate, and peak_temp is the temperature where it occurs. dtg_mean and dtg_std are the mean and standard deviation of the DTG signal. area_hemi, area_cell, and area_lig are the integrated mass losses in the 150 to 275, 275 to 400, and 400 to 600 °C windows, and peak_hemi, peak_cell, and peak_lig are the maximum rates in those same windows. ratio_cell_hemi and ratio_lig_cell are the cellulose-to-hemicellulose and lignin-to-cellulose area ratios. fwhm is the full width at half maximum of the main peak. Raw points are written DTG_T, where T is the temperature in °C.

SHAP and permutation importance answer slightly different questions. SHAP, used for the tree models, gives each feature its average contribution to a prediction using cooperative game theory, so it shows both the size and the direction of each effect^40,56^. Permutation importance, used for the other models, measures how much the held-out error grows when one feature is shuffled, so it shows how much the model relies on that feature. In both cases the features are ranked by their mean absolute effect.

For cellulose (Fig. 8a), the top features were peak_temp and the cellulose-window descriptors peak_cell and area_cell, with smaller help from ratio_lig_cell, ratio_cell_hemi and dtg_std. This makes sense: cellulose forms the sharp main peak at about 315 to 400 °C, so the height, area, and position of that peak carry its content, which agrees with earlier work on cellulose^8,47^. For hemicellulose (Fig. 8b), the top features were ratio_lig_cell, plus a group of mid- to high-temperature raw points (around DTG_447 to DTG_477), and the lignin-window descriptors area_lig and peak_lig. The model relies on ratios and scattered points rather than a single hemicellulose feature because the hemicellulose shoulder (about 200 to 315 °C) overlaps the cellulose onset and has no peak of its own. For lignin (Fig. 8c), the top features were ratio_lig_cell and peak_temp, then high-temperature raw points (DTG_617 to DTG_687) and fwhm. This fits lignin’s slow, broad breakdown and char formation at high temperature^48^. The model is using the curve’s width, not a real lignin peak.

Two cautions apply when reading these rankings. First, the features are highly correlated. Adjacent DTG points move together, and the window areas and peaks are built from the same parts of the curve. When predictors are correlated, their importance is shared, and both tree-based and unconditional permutation measures are known to be biased toward correlated variables^57^. Part of the thin, spread-out importance observed for hemicellulose and lignin is therefore due to this sharing effect, not merely a weak signal. Second, importance explains the model, not the chemistry. A feature ranks highly because the fitted model relies on it; that does not prove that the feature, on its own, determines composition. For cellulose, the ranking aligns with the known decomposition chemistry and the sharp 315 to 400 °C peak^47^, so the model is most likely using a genuine signal. For hemicellulose, and even more so for lignin, the importance is spread thinly across many weak features and ratios, and should be read alongside the modest and poor scores for those targets. The rankings describe how each model uses the available signal. They are not evidence of a strong feature-to-composition link for the weaker targets.

### Comparison with other studies

Table 3 compares our results with other machine-learning work in bioenergy. Most of these studies report high R^2^, often above 0.9, but usually for higher heating value or for gasification and pyrolysis yields, not for structural composition, and usually from a single train–test split on a fairly uniform dataset^39,42,58–63^. A fairer comparison is with studies that directly predict cellulose, hemicellulose, and lignin, most of which use spectroscopy. Those studies show two things that line up with our results.

**Table 3:** Comparison of machine-learning model performance for biomass property prediction across representative studies.

Biomass type	Method(s)	Key findings	Samples (n)	Performance (R^2^)	Refs
Diverse biomass (this work)	RF, GB, XGB, Ridge, PLS, SVR, kNN	Moderate cellulose and modest hemicellulose prediction; lignin unreliable; repeated cross-validation across feedstocks	75	Cellulose 0.50–0.56; Hemicellulose 0.38–0.43; Lignin 0.13	This study
Crop straw (NIR)	PLSR, SVR	Composition by spectroscopy; lignin the hardest	148	Cell 0.79; Hemi 0.63; Lig 0.57	^64^
Diverse biomass (FTIR)	ε-WLS-SVR (transfer)	High within-set; R^2^ falls on new feedstocks	80	Within-set > 0.95; new biomass < 0.66	^65^
Moso bamboo (NIR)	ANN, LS-SVM	Composition by spectroscopy; lignin lowest	NR	Hemi 0.92; Cell 0.91; Lig 0.89	^66^
Biomass pyrolysis (small set)	RF, XGB	Small dataset; weak holdout, some negative R^2^	small	CV 0.49–0.52; holdout R^2^ − 0.18 to 0.20	^67^
Various biomass	RF, SVM	RF best for HHV across classes	NR	RF 0.989; SVM 0.91	^59^
Mixed biomass	RF, ANN, DT	RF predicted HHV across types	NR	RF 0.989	^58^
Coal (biomass analog)	GPR, SVR, RT	High accuracy for elemental composition	NR	C 0.997; H 0.990	^60^
Biomass (proximate/ultimate)	XGB	Near-perfect HHV prediction	NR	XGB 0.999	^42^
Olive-pit biomass	LR, kNN, SVM, DT	Effective syngas H₂ prediction	NR	R^2^ 0.99	^61^
Mixed biomass	Poly-reg., DT, MLP	MLP/DT best for syngas	NR	R^2^ > 0.90	^63^
Various biomass	SVM, RF, GB	GB best for H₂ and char yield	NR	R^2^ > 0.90	^39^
Biomass–coal blend	GB	Optimised H₂ production	NR	R^2^ > 0.92	^32^
Lignocellulosic biomass	ANN	Reliable TGA-based composition	NR	R^2^ > 0.96	^8^
Pyrolytic biomass	RF, MLR	RF generalized better for bio-oil yield	NR	R^2^ 0.77	^43^
24 biomass types	CB, RF, SVM, LightGBM, kNN, XGB	CatBoost ~ 98% for H₂ yield	NR	0.985 (test)	^41^

The first rows predict structural composition (cellulose, hemicellulose, lignin); the lower rows primarily report HHV or gasification/pyrolysis yield performance. The Samples (n) column shows training-set size where reported (NR = not reported here). Smaller and within-dataset studies tend to report higher single-split scores, whereas the “this work” row reports repeated cross-validated, cross-feedstock R^2^; the small-set pyrolysis study^68^ shows how a limited dataset can give weak or negative held-out R^2^.

The first is that the three components are not as readily predictable as each other, and lignin is consistently the most difficult. Cellulose, hemicellulose, and lignin in crop straws were predicted using NIR, with R^2^ values of 0.79, 0.63, and 0.57, respectively^64^. The values obtained for cellulose, hemicellulose, and lignin from near-infrared analysis of Moso bamboo were approximately 0.91, 0.91 and 0.89, respectively^66^. Similar results are also found in other near-infrared and hyperspectral studies of biomass, where the lignin component is always the least intense of the three. This ordering is the same for all methods and feedstocks, cellulose first, then hemicellulose, then lignin. The ordering is reproduced independently, and the lignin difficulty is lower for reasons stated below, but our absolute values are lower than those reported in the above-cited literature. The difficulty of the lignin and the ordering are reproduced independently, supporting the chemistry-based explanation in Sect. "DTG behavior and its relation to composition"^46–48^, and the absolute values are lower.

Secondly, the R^2^ values are very high in those studies, and the data are typically limited to a narrow, specific range and cannot be extrapolated to other feedstocks. This is evident from a recent FTIR study. The model’s R^2^ was above 0.95 for the three components of the data but below 0.66 for new biomass, and it required a transfer-learning phase with extra samples to restore it^65^. It is the same generalization gap that our design is meant to measure, not obfuscate, using a diverse set of 75 samples and repeated nested cross-validation. Read against that gap, our cross-validated cellulose R^2^ is roughly 0.5, which is not as bad as a single-feedstock 0.9. The setting of greater interest when screening unknown samples^44,54^ is the modeling of composition across feedstocks, which here provides a more realistic estimate of DTG performance.

Sample size is also a component of the gap. The number of samples is limited (75); thus, this should yield a lower, more variable R^2^ value, regardless of the method. Research into predictive modeling has revealed that training on smaller data sets may reduce explained variance by about half^69^ compared with larger sets, since it is more difficult to separate the true signal from noise in such small data sets, resulting in greater uncertainty in the fitted model. This is especially true for biomass and bioenergy modeling, as small and overly limited datasets are used in many machine-learning studies, resulting in reduced accuracy and generalization, according to reviews^70^. All our learning curves are heading in the same direction: as indicated in Fig. 7a, the cellulose score continues to increase even after 75 samples, and does not yet seem to be leveling off. The size of the data set is therefore part of the reason for our moderate R^2^; as more samples are added, the cellulose and hemicellulose models should improve. This is further motivation to view our values as a conservative lower bound for cross-feedstock predictions, rather than the optimal values.

One more point separates our work from most of these studies. They use spectroscopy, which probes chemical bonds directly. DTG instead records thermal decomposition, an indirect and partly kinetic signal (Sect. "Effect of engineered features"), so some loss of accuracy is expected, and the lignin signal in particular is weak. Seen in this light, our results are a careful, transparent benchmark for DTG-based composition prediction across feedstocks, not a bid for the highest reported R^2^. Clearly showing that lignin cannot be reliably predicted from DTG alone is itself useful for anyone screening feedstocks.

### Synthesis of results

Separate analyses all go in the same direction. Based on the formation of DTG, the order of ease of modeling is expected to be cellulose, hemicellulose, and lignin, in accordance with their formation order. In each of the figures and tables shown below, the three components are ranked in that order; for that reason, the gains in feature engineering (Fig. 2, Table 1), the model comparison (Fig. 3, Table 2), the parity, trace, and residual plots (Figs. 4, 5 and 6), the learning curves (Fig. 7), and the feature importance (Fig. 8) are all in favour of that order. It’s because of the distinctiveness of each component in the curve. Cellulose shows a sharp peak and is moderately and consistently well predicted (R^2^ is about 0.50–0.56). Hemicellulose overlaps with cellulose and is expected to be moderate (0.38–0.43). No specific peak exists for lignin, and it can not be predicted reliably (about 0.13).

There are two points of this agreement which give it weight. First, there is an ordering of seven algorithms, ranging from linear to kernel methods to tree ensembles, which all agree on. That convergence is not with respect to the selection of model; it is with respect to the information in the DTG signal^51^. Second, in independent studies of composition using different instruments and feedstocks, lignin is always the most difficult fraction^64,66^. If multiple lines of evidence agree, the result is more robust than any single line of evidence. The two validation schemes share the same picture. The prediction agrees for cellulose and hemicellulose, two components that can be predicted, and disagrees only for lignin, the one that cannot be predicted, which is where an unstable estimator shows up^52,54^. So, the result refers to the measured object, rather than the model.

In practice, DTG can serve as a quick proxy for cellulose and, to a lesser degree, hemicellulose, but not for lignin without an additional measurement. The reliable range is also the well-sampled one. The predictions can be trusted for common mid-range compositions, but should not be extended to sparse, high-lignin char samples that lie outside the region on which the model was built^53^.

### Limitations

A few limits apply, and it is worth being precise about each. The dataset has 75 samples. As set out in Sect. "Comparison with other studies", a set of this size is expected to yield lower and more variable R^2^ than a larger one^69,70^, and the cellulose learning curve (Fig. 7a) is still rising, so more samples, especially more high-lignin references, would likely raise the scores. They would also test the extrapolation that now fails: the high-lignin char samples lie at the sparse edge of the data, so predictions there are extrapolations beyond the applicability domain and are the least reliable^53^.

The dataset is also not fully independent. The blends share features with their parent materials, so some samples are correlated rather than independent draws. When data exhibit this kind of dependence structure, ordinary random cross-validation ignores it. It tends to underestimate the true error because near-duplicate samples can fall into both the training and test folds^71^. Our repeated and nested cross-validation reduces this effect but does not remove it. A stricter leave-one-feedstock-out scheme, in which all samples of a feedstock are held out together, would give a more conservative and more realistic estimate and is the natural next test^71^.

DTG alone also provides little lignin-specific information because lignin decomposes slowly over a wide range and leaves no distinct peak^46–48^. This is a limit of the measurement, not of the model, so the most promising route is to add a second, more direct probe. Spectroscopic methods such as NIR and FTIR resolve lignin more accurately and achieve higher compositional accuracy^64–66^, so pairing DTG with a spectroscopic input is a sensible way to improve lignin estimates. Finally, the results are specific to the feedstocks, instrument, and protocol used here. Performance can decline when a model trained on one set of feedstocks encounters new ones^65^, so the scores should be rechecked and the model recalibrated, if needed, before use with other materials or instruments.

## Conclusions

DTG has moderately high cross-validated accuracy for cellulose (R^2^ ~ 0.50 to 0.56) and moderately low cross-validated accuracy for hemicellulose (R^2^ ~ 0.38 to 0.43) across 75 biomass samples from various feedstocks. Lignin, on the other hand, was not predictable with a consistent R^2^ (about 0.13). All descriptors were enhanced with the engineered descriptors. The ranking of the three components and the difficulty of lignin follows from the degree of clarity of the signature they leave in the curve, and it is the same ranking that emerges in independent studies of composition. Repeated and nested cross-validation was necessary: a high lignin score in a single pass did not hold up, which is an optimistic result that can be obtained with a small, varied dataset in a single pass.

The contribution is a thoughtful, multi-feedstock benchmark and not a big headline R^2^. DTG has been tested on a variety of feedstocks, and is a useful rapid screening method for cellulose, of some utility for hemicellulose, and not yet reliable for lignin. Three steps can be taken from that. The scores should increase and stabilize with larger, more balanced data sets and more high-lignin samples. A leave-one-feedstock-out validation will provide a more stringent and realistic estimate of the effects of correlation and blending in the data. A combination of DTG with another direct probe, such as NIR or FTIR spectroscopy, is the most promising approach for obtaining a reliable estimate of lignin. The clear statement of these limits and an open, reproducible pipeline gives a transparent baseline and a foundation on which these steps could proceed.

## Supplementary Information

Below is the link to the electronic supplementary material.

The following supporting information is provided as a separate Supplementary file (document and spreadsheet):

Supplementary Material 1
Table S1. List of 75 biomass samples, with feedstock group and measured cellulose, hemicellulose, and lignin contents. Table S2—definitions of the 13 engineered DTG descriptors and their temperature windows. Table S3. Full per-model results for both feature sets (raw 67 vs raw + engineered): R2, RMSE and MAE for all seven algorithms, with the repeated fivefold uncertainty. Table S4. Survey of recent machine-learning work in bioenergy cited in the Introduction. Dataset S1 (spreadsheet). The full numerical dataset includes the 67-point DTG matrix, the three measured compositions for all 75 samples, the engineered-feature matrix, and the full results and uncertainty tables.

Supplementary Material 2

## Data Availability

The datasets used and/or analysed during the current study available from the corresponding author on reasonable request.

## List of symbols

C: Penalty (regularisation) parameter of support-vector regression
k: Number of nearest neighbors (kNN); number of folds in k-fold cross-validation
MAE: Mean absolute error (%)
N: Number of samples
R^2^: Coefficient of determination
RMSE: Root-mean-square error (%)
T: Temperature (°C)
${y}_{i}$: Measured composition of sample i (%)
$\overline{{y }_{i}}$: Predicted composition of sample i (%)
$\overline{y }$: Mean of the measured compositions (%)
α: L2 regularisation strength (ridge regression)
β: Heating rate (K min^−1^)
γ: Kernel coefficient (support-vector regression)
λ: L2 penalty term, reg_lambda (XGBoost)
i: Sample index

## Abbreviations

ANN: Artificial neural network
ASTM: American society for testing and materials
CatBoost: Categorical boosting
CV: Cross-validation
DT: Decision tree
DTG: Derivative thermogravimetric
FTIR: Fourier-transform infrared
FWHM: Full width at half maximum
GAN: Generative adversarial network
GB: Gradient boosting
GPR: Gaussian process regression
HHV: Higher heating value
HPLC: High-performance liquid chromatography
kNN: K-nearest neighbors
LightGBM: Light gradient-boosting machine
XGB: Extreme gradient boosting
LOO-CV: Leave-one-out cross-validation
LR: Linear regression
L2: L2 regularisation (ridge/squared-magnitude penalty)
MLP: Multilayer perceptron
MLR: Multiple linear regression
MSE: Mean squared error
NIR: Near-infrared (spectroscopy)
NREL: National renewable energy laboratory
PLS: Partial least squares (regression)
RF: Random forest
SVM: Support vector machine
SVR: Support-vector regression
RT: Regression tree
SD: Standard deviation
SHAP: SHapley Additive exPlanations
TGA: Thermogravimetric analysis

## Code availability

The analysis code and dataset are available at 10.5281/zenodo.21130334.

## References

[1] Pattanayak, S., Loha, C., Hauchhum, L. & Sailo, L. Application of MLP-ANN models for estimating the higher heating value of bamboo biomass. *Biomass Convers. Biorefinery* **11**, 2499–2508. 10.1007/s13399-020-00685-2 (2021).

[2] Paul, S. & Dutta, A. Challenges and opportunities of lignocellulosic biomass for anaerobic digestion. *Resour. Conserv. Recycl.* **130**, 164–174. 10.1016/j.resconrec.2017.12.005 (2018).

[3] R. Krishnan, L. Hauchhum, R. Gupta, S. Pattanayak, Prediction of Equations for Higher Heating Values of Biomass Using Proximate and Ultimate Analysis. In: 2nd Int. Conf. Energy, Power Environ. Towar. Smart Technol. ICEPE 2018. 10.1109/EPETSG.2018.8658984. (2018)

[4] Nhuchhen, D. R. & Afzal, M. T. HHV predicting correlations for torrefied biomass using proximate and ultimate analyses. *Bioengineering* **4**, 7. 10.3390/bioengineering4010007 (2017). 28952487 10.3390/bioengineering4010007 PMC5590445

[5] Ullah, K. et al. Assessing the lignocellulosic biomass resources potential in developing countries: A critical review. *Renew. Sustain. Energy Rev.* **51**, 682–698. 10.1016/j.rser.2015.06.044 (2015).

[6] Saha, D., Roy, B. & Kundu, P. P. Hydrogen induction in a dual-fuel mode common rail direct injection compression ignition engine running on ethanol–diesel blends: Combustion, performance, and emission characteristics. *J. Energy Resources Technol. Part A: Sustain. Renew. Energy*10.1115/1.4068211 (2025).

[7] Saha, D., Roy, B. & Kundu, P. P. Influence of injection timing variation on combustion-emission-performance aspects of emulsified plastic oil-run compression ignition engine. *J. Energy Resour. Technol. Trans. ASME* **146**, 1–9. 10.1115/1.4065540 (2024).

[8] Carrier, M. et al. Thermogravimetric analysis as a new method to determine the lignocellulosic composition of biomass. *Biomass Bioenergy* **35**, 298–307. 10.1016/j.biombioe.2010.08.067 (2011).

[9] Scheller, H. V. & Ulvskov, P. Hemicelluloses. *Annu. Rev. Plant Biol.* **61**, 263–289 (2010). 20192742 10.1146/annurev-arplant-042809-112315

[10] Sengupta, D. & Pike, R. W. *Chemicals from Biomass: Integrating Bioprocesses into Chemical Production Complexes for Sustainable Development* (CRC Press, 2012).

[11] Singh, K., Risse, M., Das, K. C. & Worley, J. Determination of composition of cellulose and lignin mixtures using thermogravimetric analysis. *J. Energy Resour. Technol. Trans. ASME* **131**, 0222011–0222016. 10.1115/1.3120349 (2009).

[12] Gurram, R. et al. Technical possibilities of bioethanol production from coffee pulp: A renewable feedstock. *Clean Technol. Environ. Policy* **18**, 269–278 (2016).

[13] Nimmanterdwong, P., Chalermsinsuwan, B. & Piumsomboon, P. Prediction of lignocellulosic biomass structural components from ultimate/proximate analysis. *Energy* **222**, 119945 (2021).

[14] Alzate, C. A. C. & Toro, O. J. S. Energy consumption analysis of integrated flowsheets for production of fuel ethanol from lignocellulosic biomass. *Energy* **31**, 2447–2459 (2006).

[15] Naik, S., Goud, V. V., Rout, P. K., Jacobson, K. & Dalai, A. K. Characterization of Canadian biomass for alternative renewable biofuel. *Renew. Energy* **35**, 1624–1631 (2010).

[16] Parikh, J., Channiwala, S. A. & Ghosal, G. K. A correlation for calculating elemental composition from proximate analysis of biomass materials. *Fuel* **86**, 1710–1719 (2007).

[17] Raj, T. et al. Physical and chemical characterization of various Indian agriculture residues for biofuels production. *Energy Fuels* **29**, 3111–3118 (2015).

[18] Coffey, D. G., Bell, D. A. & Henderson, A. *Cellulose and Cellulose Derivatives* (Marcel Dekker Inc., 1995).

[19] Hayes, M. H. B., Mylotte, R. & Swift, R. S. Humin: its composition and importance in soil organic matter. *Adv. Agron.* **143**, 47–138 (2017).

[20] Stefanidis, S. D. et al. A study of lignocellulosic biomass pyrolysis via the pyrolysis of cellulose, hemicellulose and lignin. *J. Anal. Appl. Pyrolysis* **105**, 143–150 (2014).

[21] TAPPI, T. Carbohydrate composition of extractive-free wood and wood pulp by gas-liquid chromatography, TAPPI Test Method T249 C. (1985).

[22] Effland, M.J. Modified procedure to determine acid-insoluble lignin in wood and pulp, Tappi; (United States) 60 (1977).

[23] Sluiter, A. et al. Determination of structural carbohydrates and lignin in biomass. *Lab. Anal. Proced.* **1617**, 1–16 (2008).

[24] Umenweke, G. C., Afolabi, I. C., Epelle, E. I. & Okolie, J. A. Machine learning methods for modeling conventional and hydrothermal gasification of waste biomass: A review. *Bioresour. Technol. Reports* **17**, 100976. 10.1016/j.biteb.2022.100976 (2022).

[25] Liu, J., Ulishney, C. & Dumitrescu, C. E. Random forest machine learning model for predicting combustion feedback information of a natural gas spark ignition engine. *J. Energy Resour. Technol. Trans. ASME* **143**, 1–7. 10.1115/1.4047761 (2021).

[26] Sharma, V. et al. Downdraft gasification for biogas production: The role of artificial intelligence. *J. Energy Resour. Technol.* **146**, 1–13. 10.1115/1.4066059 (2024).

[27] Petro, N. & Lopez, F. Machine learning-based digital twins reduce seasonal remapping in aeroderivative gas turbines. *J. Energy Resour. Technol. Trans. ASME* **144**, 1–6. 10.1115/1.4052994 (2022).

[28] Vargas, G. G., Ortiz, P. S. & de Oliveira, S. Performance analysis of waste biomass gasification and renewable hydrogen production by neural network algorithm. *J. Energy Resour. Technol. Trans. ASME* **146**, 1–12. 10.1115/1.4064849 (2024).

[29] Ishaq, H. & Dincer, I. An efficient energy utilization of biomass energy-based system for renewable hydrogen production and storage. *J. Energy Resour. Technol. Trans. ASME* **144**, 1–11. 10.1115/1.4050875 (2022).

[30] Shafizadeh, A. et al. Machine learning predicts and optimizes hydrothermal liquefaction of biomass. *Chem. Eng. J.* **445**, 136579 (2022).

[31] Ascher, S., Wang, X., Watson, I., Sloan, W. & You, S. Interpretable machine learning to model biomass and waste gasification. *Bioresour. Technol.* **364**, 128062. 10.1016/j.biortech.2022.128062 (2022). 36202285 10.1016/j.biortech.2022.128062

[32] Pan, J. et al. Machine learning optimization for enhanced biomass-coal co-gasification. *Renew. Energy* **229**, 120772. 10.1016/j.renene.2024.120772 (2024).

[33] Li, H. et al. Machine-learning-aided thermochemical treatment of biomass: A review. *Biofuel Res. J.* **10**, 1786–1809. 10.18331/BRJ2023.10.1.4 (2023).

[34] Afolabi, I. C., Epelle, E. I., Gunes, B., Güleç, F. & Okolie, J. A. Data-driven machine learning approach for predicting the higher heating value of different biomass classes. *Clean Technol.* **4**, 1227–1241 (2022).

[35] Akinpelu, D. A., Adekoya, O. A., Oladoye, P. O., Ogbaga, C. C. & Okolie, J. A. Machine learning applications in biomass pyrolysis: from biorefinery to end-of-life product management. *Digit. Chem. Eng.* **8**, 100103 (2023).

[36] Sison, A. E., Etchieson, S. A., Güleç, F., Epelle, E. I. & Okolie, J. A. Process modelling integrated with interpretable machine learning for predicting hydrogen and char yield during chemical looping gasification. *J. Clean. Prod.* **414**, 137579 (2023).

[37] Lundberg, S.M. and Lee, S.I. A unified approach to interpreting model predictions, in: Adv. Neural Inf. Process. Syst., pp. 4766–4775. (2017).

[38] Ozveren, U. An artificial intelligence approach to predict gross heating value of lignocellulosic fuels. *J. Energy Inst.* **90**, 397–407 (2017).

[39] Ascher, S., Wang, X., Watson, I., Sloan, W. & You, S. Interpretable machine learning to model biomass and waste gasification. *Bioresour. Technol.* **364**, 128062 (2022). 36202285 10.1016/j.biortech.2022.128062

[40] Ascher, S., Watson, I. & You, S. Machine learning methods for modelling the gasification and pyrolysis of biomass and waste. *Renew. Sustain. Energy Rev.* **155**, 111902 (2022).

[41] Tuntiwongwat, T., Thammawiset, S., Srinophakun, T. R., Ngamcharussrivichai, C. & Sukpancharoen, S. BCLH2Pro: A novel computational tools approach for hydrogen production prediction via machine learning in biomass chemical looping processes. *Energy AI* **18**, 100414. 10.1016/j.egyai.2024.100414 (2024).

[42] Dai, Z. et al. Machine learning prediction of higher heating value of biomass. *Biomass Convers. Biorefinery* **13**, 3659–3667. 10.1007/s13399-021-01273-8 (2023).

[43] Tang, Q. et al. Prediction of bio-oil yield and hydrogen contents based on machine learning method: Effect of biomass compositions and pyrolysis conditions. *Energy Fuels* **34**, 11050–11060. 10.1021/acs.energyfuels.0c01893 (2020).

[44] Kartal, F. & Özveren, U. An improved machine learning approach to estimate hemicellulose, cellulose, and lignin in biomass. *Carbohydr. Polym. Technol. Appl.*10.1016/j.carpta.2021.100148 (2021).

[45] and D.C. A. Sluiter, B. Hames, R. Ruiz, C. Scarlata, J. Sluiter, D. Templeton, Biomass Compositional Analysis Laboratory Procedures | Bioenergy | NREL, NREL (2012). https://www.nrel.gov/bioenergy/biomass-compositional-analysis.html (accessed July 22, 2024).

[46] Font, R. Decomposition of organic wastes: Thermal analysis and evolution of volatiles. *Handb. Therm. Anal. Calorim.* **6**, 339–397. 10.1016/B978-0-444-64062-8.00001-2 (2018).

[47] Escalante, J. et al. Pyrolysis of lignocellulosic, algal, plastic, and other biomass wastes for biofuel production and circular bioeconomy: A review of thermogravimetric analysis (TGA) approach. *Renew. Sustain. Energy Rev.* **169**, 112914. 10.1016/J.RSER.2022.112914 (2022).

[48] Ong, H. C. et al. Variation of lignocellulosic biomass structure from torrefaction: A critical review. *Renew. Sustain. Energy Rev.* **152**, 111698. 10.1016/J.RSER.2021.111698 (2021).

[49] Aghbashlo, M. et al. Machine learning technology in biodiesel research: A review. *Prog. Energy Combust. Sci.* **85**, 100904. 10.1016/J.PECS.2021.100904 (2021).

[50] T. Chen, C. Guestrin, XGBoost: A scalable tree boosting system, in: Proc. ACM SIGKDD Int. Conf. Knowl. Discov. Data Min., 2016. 10.1145/2939672.2939785.

[51] Wolpert, D. H. & Macready, W. G. No free lunch theorems for optimization. *IEEE Trans. Evol. Comput.* **1**, 67–82. 10.1109/4235.585893 (1997).

[52] Austin, G. I., Pe’er, I. & Korem, T. Distributional bias compromises leave-one-out cross-validation. *Sci. Adv.* **11**, eadx6976. 10.1126/sciadv.adx6976 (2025). 41313770 10.1126/sciadv.adx6976 PMC12662204

[53] Sahigara, F. et al. Comparison of different approaches to define the applicability domain of QSAR models. *Molecules* **17**, 4791–4810. 10.3390/molecules17054791 (2012). 22534664 10.3390/molecules17054791 PMC6268288

[54] Varma, S. & Simon, R. Bias in error estimation when using cross-validation for model selection. *BMC Bioinformatics* **7**, 91. 10.1186/1471-2105-7-91 (2006). 16504092 10.1186/1471-2105-7-91 PMC1397873

[55] Hastie, T., Tibshirani, R. & Friedman, J. *The Elements of Statistical Learning: Data Mining, Inference, and Prediction* 2nd ed. (Springer, 2009).

[56] Lundberg, S. M. et al. From local explanations to global understanding with explainable AI for trees. *Nat. Mach. Intell.* **2**, 56–67. 10.1038/s42256-019-0138-9 (2020). 32607472 10.1038/s42256-019-0138-9 PMC7326367

[57] Strobl, C., Boulesteix, A.-L., Kneib, T., Augustin, T. & Zeileis, A. Conditional variable importance for random forests. *BMC Bioinformatics* **9**, 307. 10.1186/1471-2105-9-307 (2008). 18620558 10.1186/1471-2105-9-307 PMC2491635

[58] Dubey, R. & Guruviah, V. Machine learning approach for categorical biomass higher heating value prediction based on proximate analysis. *Energy Sources, Part A: Recovery, Utiliz., Environ. Effects* **44**, 3381–3394. 10.1080/15567036.2022.2065386 (2022).

[59] Brandić, I. et al. Comparison of different machine learning models for modelling the higher heating value of biomass. *Mathematics*10.3390/math11092098 (2023).

[60] Ceylan, Z. & Sungur, B. Estimation of coal elemental composition from proximate analysis using machine learning techniques. *Energy Sources, Part A: Recovery, Utiliz. Environ. Effects* **42**, 2576–2592. 10.1080/15567036.2020.1790696 (2020).

[61] Ozbas, E. E., Aksu, D., Ongen, A., Aydin, M. A. & Ozcan, H. K. Hydrogen production via biomass gasification, and modeling by supervised machine learning algorithms. *Int. J. Hydrogen Energy* **44**, 17260–17268. 10.1016/j.ijhydene.2019.02.108 (2019).

[62] Olca, K. D. & Yücel, Ö. Unveiling the potential of operating time in improving machine learning models’ performance for waste biomass gasification systems. *Renew. Energy*10.1016/j.renene.2024.121621 (2024).

[63] Elmaz, F., Yücel, Ö. & Mutlu, A. Y. Predictive modeling of biomass gasification with machine learning-based regression methods. *Energy*10.1016/j.energy.2019.116541 (2020).

[64] Zhao, Y. et al. Predictive modeling of lignocellulosic content in crop straws using NIR spectroscopy. *Plants* **14**, 1430. 10.3390/plants14101430 (2025). 40430995 10.3390/plants14101430 PMC12114956

[65] Li, J. et al. Rapid prediction of cellulose, hemicellulose, and lignin content in biomass using FTIR based on transfer learning. *J. Clean. Prod.*10.1016/j.jclepro.2026.147656 (2026).

[66] Li, X., Sun, C., Zhou, B. & He, Y. Determination of hemicellulose, cellulose and lignin in Moso bamboo by near infrared spectroscopy. *Sci. Rep.* **5**, 17210. 10.1038/srep17210 (2015). 26601657 10.1038/srep17210 PMC4658639

[67] Asif, M. et al. Machine learning-based prediction of biomass pyrolysis kinetics: integrating mechanistic modeling and compositional features. *RSC Adv.* **16**, 28036–28047. 10.1039/D6RA01011C (2026). 42205268 10.1039/d6ra01011c PMC13202439

[68] Vassilev, S. V., Baxter, D., Andersen, L. K. & Vassileva, C. G. An overview of the chemical composition of biomass. *Fuel* **89**(5), 913–933. 10.1016/j.fuel.2009.10.022 (2010).

[69] Riley, R. D. et al. Importance of sample size on the quality and utility of AI-based prediction models for healthcare. *Lancet Digit. Health* **7**, 100857. 10.1016/j.landig.2025.01.013 (2025). 40461350 10.1016/j.landig.2025.01.013

[70] Wang, R. et al. Enhancing biomass conversion to bioenergy with machine learning: Gains and problems. *Sci. Total Environ.* **927**, 172310. 10.1016/j.scitotenv.2024.172310 (2024). 38599406 10.1016/j.scitotenv.2024.172310

[71] Roberts, D. R. et al. Cross-validation strategies for data with temporal, spatial, hierarchical, or phylogenetic structure. *Ecography* **40**, 913–929. 10.1111/ecog.02881 (2017).

