# Supplementary material for: Machine-learning prediction of biomass chemical composition using derivative thermogravimetric data of different biomass feedstocks
Source: Sci Rep. 2026 Aug 12;16:25132. doi: 10.1038/s41598-026-65442-3 (PMC13469630; doi:10.1038/s41598-026-65442-3)
Supplement: Supplementary file 1 [file 41598_2026_65442_MOESM2_ESM.docx]

**Supplementary Material**

*Machine-Learning Prediction of Biomass Chemical Composition Using Derivative Thermogravimetric Data of Different Biomass Feedstocks*

This document provides supporting tables. The complete numerical dataset (DTG feature matrix, engineered features, and full results) is provided in the accompanying spreadsheet, Dataset S1 (Supplementary_Data_S1.xlsx).

# Table S1. Biomass samples and measured composition

*Cellulose, hemicellulose and lignin contents (wt %, dry basis) for the 75 samples used as regression targets. Feedstock groups are inferred from sample identifiers and should be verified by the authors.*

| **#** | **Sample ID** | **Feedstock group** | **Cellulose** | **Hemicellulose** | **Lignin** | **Data Ref.** |
| --- | --- | --- | --- | --- | --- | --- |
| 1 | B_1 | Dendrocalamus hamiltonii | 46.1 | 17.6 | 24.8 | Experimental |
| 2 | B_2 | Schizostachyum dulloa | 43.2 | 22.4 | 25.5 | Experimental |
| 3 | B_3 | Bambusa nutans Wall. Ex Munro | 42.5 | 21.3 | 26.2 | Experimental |
| 4 | B_4 | Dendrocalamus asper | 41.7 | 18.8 | 28.1 | Experimental |
| 5 | B_5 | Sasa Fortunei | 41.2 | 21.5 | 23.4 | Experimental |
| 6 | B_6 | Bambusa nutans | 42.0 | 21.1 | 25.8 | Experimental |
| 7 | B_7 | Pseudosasa Japonica | 45.3 | 19.2 | 25.3 | Experimental |
| 8 | B_8 | Dendrocalamus sikkimensis Gamble | 43.6 | 18.7 | 26.1 | Experimental |
| 9 | B_9 | Dendrocalamus giganteus Munro | 43.5 | 20.8 | 27.4 | Experimental |
| 10 | B_10 | Sinarundinaria falcate (Nees) | 40.9 | 21.7 | 24.9 | Experimental |
| 11 | B_11 | Thrysostachys oliveri Gamble | 42.7 | 20.3 | 25.0 | Experimental |
| 12 | B_12 | Bambusa multiplex (Lour.) | 41.8 | 22.0 | 24.6 | Experimental |
| 13 | B_13 | Bambusa balcooa Roxb | 44.1 | 19.5 | 26.3 | Experimental |
| 14 | B_14 | Bambusa Alemtemshii Naithani | 42.3 | 21.6 | 24.9 | Experimental |
| 15 | B_15 | Bambusa Vulgaris var. Striata | 44.8 | 19.2 | 24.6 | Experimental |
| 16 | B_16 | Chimonobambusa callosal Munro | 43.9 | 18.4 | 25.2 | Experimental |
| 17 | B_17 | Bambusa Vulgaris cv. Wamin (Brandis) McClure | 45.1 | 19.1 | 25.1 | Experimental |
| 18 | B_18 | Bambusa bambos (Linn.) | 43.3 | 20.2 | 26.5 | Experimental |
| 19 | B_19 | Melocanna baccifera | 52.8 | 21.2 | 25.2 | Experimental |
| 20 | B_20 | Oxytenanthera Albociliata Munro | 43.4 | 19.8 | 24.7 | Experimental |
| 21 | B_21 | Dendrocalamus Longispathus Kurz | 44.2 | 18.7 | 25.6 | Experimental |
| 22 | BN_Shell | Betel nut shell | 64.0 | 10.0 | 17.0 | Experimental |
| 23 | R_Straw | Rice (straw/husk) | 39.0 | 24.0 | 21.0 | Experimental |
| 24 | R10_Husk | Rice (straw/husk) | 39.0 | 21.0 | 17.0 | Experimental |
| 25 | R20_Husk | Rice (straw/husk) | 42.0 | 23.0 | 24.0 | Experimental |
| 26 | R30_Husk | Rice (straw/husk) | 46.0 | 21.0 | 27.0 | Experimental |
| 27 | R50_Husk | Rice (straw/husk) | 38.0 | 26.0 | 22.0 | Experimental |
| 28 | R_Husk | Rice (straw/husk) | 42.0 | 20.0 | 13.0 | Experimental |
| 29 | B25_RS75 | Blend | 40.5 | 21.8 | 19.0 | Experimental |
| 30 | B50_RS50 | Blend | 42.0 | 22.5 | 21.0 | Experimental |
| 31 | B75_RS25 | Blend | 43.5 | 23.3 | 23.0 | Experimental |
| 32 | B25_BN75 | Blend | 59.3 | 13.5 | 19.0 | Experimental |
| 33 | B50_BN50 | Blend | 54.5 | 17.0 | 21.0 | Experimental |
| 34 | B75_BN25 | Blend | 49.8 | 20.5 | 23.0 | Experimental |
| 35 | B25_RH75 | Blend | 13.2 | 5.8 | 7.4 | Experimental |
| 36 | B50_RH50 | Blend | 21.7 | 11.3 | 13.8 | Experimental |
| 37 | B75_RH25 | Blend | 35.6 | 17.4 | 19.1 | Experimental |
| 38 | B25_CC75 | Blend | 10.8 | 7.1 | 8.2 | Experimental |
| 39 | B50_CC50 | Blend | 24.3 | 13.7 | 11.6 | Experimental |
| 40 | B75_CC25 | Blend | 32.5 | 16.2 | 21.4 | Experimental |
| 41 | B25_PNS75 | Blend | 14.5 | 6.3 | 6.9 | Experimental |
| 42 | B50_PNS50 | Blend | 23.6 | 10.8 | 14.2 | Experimental |
| 43 | B75_PNS25 | Blend | 31.8 | 19.1 | 17.6 | Experimental |
| 44 | MB | Biomass (other) | 40.0 | 22.0 | 23.0 | Experimental |
| 45 | RML | Biomass (other) | 30.0 | 25.0 | 25.0 | Experimental |
| 46 | RMLC_400 | Char / residue | 18.0 | 9.0 | 52.0 | Experimental |
| 47 | RMLC_500 | Char / residue | 11.0 | 5.0 | 65.0 | Experimental |
| 48 | RMLC_600 | Char / residue | 6.0 | 2.0 | 78.0 | Experimental |
| 49 | CC | corn cob | 41.5 | 34.7 | 15.2 | 74-77, 86,87 |
| 50 | EW | eucalyptus wood | 41.4 | 35.3 | 23.3 | 74-77, 87 |
| 51 | PNS | peanut shell | 37.0 | 18.0 | 29.0 | 74-77, 90 |
| 52 | CS | cotton stalk | 34.0 | 22.0 | 36.0 | 74-77 |
| 53 | C_stover | corn stover | 39.0 | 19.1 | 15.1 | 74-77, 85 |
| 54 | CSW | Coconut Shell waste | 38.0 | 26.0 | 24.0 | 74-77, 94 |
| 55 | WS | wheat straw | 38.0 | 27.0 | 20.0 | 74-77, 91 |
| 56 | PKS | palm kernel shell | 28.0 | 22.0 | 45.0 | 74-77, 84 |
| 57 | P0W10 | 100% Wood Sawdust Pellet | 44.0 | 25.0 | 28.0 | 74-77, 92,93 |
| 58 | P5W5 | 50% Poplar Wood + 50% Tea Waste Pellet | 40.5 | 21.5 | 28.5 | 74-77, 92, 93 |
| 59 | P3W7 | 30% Poplar Wood + 70% Tea Waste Pelle | 41.9 | 22.9 | 28.3 | 74-77, 92, 93 |
| 60 | P7W3 | 70% Poplar Wood + 30% Tea Waste Pellet | 39.1 | 20.1 | 28.7 | 74-77, 92, 93 |
| 61 | P10W0 | 100% Poplar Wood Pellet | 37.0 | 18.0 | 29.0 | 74-77, 92, 93 |
| 62 | GNS | groundnut shell | 26.30 | 19.50 | 38.30 | 74-77, 88 |
| 63 | CPS | Cowpea Shell | 21.30 | 21.50 | 28.40 | 74-77, 88 |
| 64 | PW | poplar wood | 46.0 | 22.0 | 24.0 | 74-77, 93 |
| 65 | ACG | *Axonopus compressus* Grass | 36.0 | 30.0 | 18.0 | 74-77, 83 |
| 66 | MS | Miscanthus Straw | 43.0 | 27.0 | 22.0 | 74-77, 82 |
| 67 | NGT | Napier Grass (Stem + Leaf) | 39.0 | 29.0 | 17.0 | 74-77, 81 |
| 68 | NGS | Napier Grass Stem | 42.0 | 27.0 | 18.0 | 74-77, 81 |
| 69 | NGL | Napier Grass Leaf | 35.0 | 31.0 | 15.0 | 74-77, 81 |
| 70 | JS | Jute Stick | 45.0 | 22.0 | 26.0 | 74-77, 79 |
| 71 | CP | Coffee Pulp | 29.0 | 24.0 | 27.0 | 74-77, 79 |
| 72 | CH | Coffee Husk | 35.0 | 31.0 | 24.0 | 74-77, 79 |
| 73 | TW | Tea Waste | 24.0 | 18.0 | 31.0 | 74-77, 93 |
| 74 | PR | Pineapple Residue | 38.0 | 29.0 | 18.0 | 74-77, 78 |
| 75 | BPS | Banana Pseudostem | 39.0 | 28.0 | 16.0 | 74-77, 78 |

# Table S2. Definitions of the engineered DTG descriptors

*The 13 physically interpretable features computed from each sample's DTG curve, in addition to the 67 raw DTG points (DTG_T, where T is temperature in degrees Celsius).*

| **Feature** | **Definition / physical meaning** |
| --- | --- |
| peak_height | Maximum mass-loss rate of the DTG curve |
| peak_temp | Temperature of the maximum mass-loss rate |
| dtg_mean | Mean of the DTG signal over all temperatures |
| dtg_std | Standard deviation of the DTG signal (overall variability) |
| area_hemi | Integrated mass loss in the 150–275 °C window (hemicellulose regime) |
| peak_hemi | Maximum mass-loss rate within 150–275 °C |
| area_cell | Integrated mass loss in the 275–400 °C window (cellulose regime) |
| peak_cell | Maximum mass-loss rate within 275–400 °C |
| area_lig | Integrated mass loss in the 400–600 °C window (lignin/char regime) |
| peak_lig | Maximum mass-loss rate within 400–600 °C |
| ratio_cell_hemi | Ratio of cellulose-window area to hemicellulose-window area |
| ratio_lig_cell | Ratio of lignin-window area to cellulose-window area |
| fwhm | Full width at half maximum of the main decomposition peak |

# Table S3. Full per-model results (nested leave-one-out CV)

*Held-out R², RMSE and MAE for all seven algorithms and both feature sets (raw 67 points vs raw + engineered), for each component. The best model per component (engineered set) is reported in the main-text Table 2.*

| **Feature set** | **Component** | **Model** | **R²** | **RMSE (%)** | **MAE (%)** |
| --- | --- | --- | --- | --- | --- |
| Raw DTG (67) | Cellulose | RF | 0.456 | 7.63 | 5.15 |
| Raw DTG (67) | Cellulose | GB | 0.488 | 7.41 | 5.22 |
| Raw DTG (67) | Cellulose | XGB | 0.490 | 7.39 | 5.23 |
| Raw DTG (67) | Cellulose | Ridge | -0.183 | 11.26 | 6.42 |
| Raw DTG (67) | Cellulose | PLS | 0.292 | 8.71 | 6.12 |
| Raw DTG (67) | Cellulose | SVR | 0.500 | 7.32 | 5.18 |
| Raw DTG (67) | Cellulose | kNN | 0.337 | 8.43 | 5.75 |
| Raw DTG (67) | Hemicellulose | RF | 0.321 | 5.27 | 3.77 |
| Raw DTG (67) | Hemicellulose | GB | 0.229 | 5.62 | 3.89 |
| Raw DTG (67) | Hemicellulose | XGB | 0.208 | 5.69 | 3.89 |
| Raw DTG (67) | Hemicellulose | Ridge | -0.241 | 7.13 | 4.46 |
| Raw DTG (67) | Hemicellulose | PLS | -0.546 | 7.95 | 4.72 |
| Raw DTG (67) | Hemicellulose | SVR | 0.421 | 4.87 | 3.58 |
| Raw DTG (67) | Hemicellulose | kNN | 0.370 | 5.08 | 3.57 |
| Raw DTG (67) | Lignin | RF | 0.058 | 10.19 | 6.04 |
| Raw DTG (67) | Lignin | GB | -0.134 | 11.19 | 6.04 |
| Raw DTG (67) | Lignin | XGB | 0.087 | 10.04 | 5.70 |
| Raw DTG (67) | Lignin | Ridge | 0.048 | 10.25 | 5.61 |
| Raw DTG (67) | Lignin | PLS | -0.060 | 10.81 | 6.22 |
| Raw DTG (67) | Lignin | SVR | 0.399 | 8.15 | 5.03 |
| Raw DTG (67) | Lignin | kNN | 0.019 | 10.40 | 5.58 |
| Raw + engineered | Cellulose | RF | 0.574 | 6.76 | 4.78 |
| Raw + engineered | Cellulose | GB | 0.415 | 7.92 | 5.52 |
| Raw + engineered | Cellulose | XGB | 0.563 | 6.84 | 5.12 |
| Raw + engineered | Cellulose | Ridge | 0.066 | 10.00 | 5.97 |
| Raw + engineered | Cellulose | PLS | 0.359 | 8.29 | 5.72 |
| Raw + engineered | Cellulose | SVR | 0.502 | 7.31 | 5.17 |
| Raw + engineered | Cellulose | kNN | 0.544 | 6.99 | 5.23 |
| Raw + engineered | Hemicellulose | RF | 0.410 | 4.91 | 3.66 |
| Raw + engineered | Hemicellulose | GB | 0.165 | 5.84 | 4.03 |
| Raw + engineered | Hemicellulose | XGB | 0.267 | 5.48 | 3.86 |
| Raw + engineered | Hemicellulose | Ridge | -0.093 | 6.69 | 4.14 |
| Raw + engineered | Hemicellulose | PLS | -0.373 | 7.49 | 4.30 |
| Raw + engineered | Hemicellulose | SVR | 0.418 | 4.88 | 3.61 |
| Raw + engineered | Hemicellulose | kNN | 0.447 | 4.76 | 3.46 |
| Raw + engineered | Lignin | RF | 0.448 | 7.81 | 4.82 |
| Raw + engineered | Lignin | GB | 0.470 | 7.65 | 4.65 |
| Raw + engineered | Lignin | XGB | 0.480 | 7.57 | 4.70 |
| Raw + engineered | Lignin | Ridge | 0.545 | 7.09 | 4.51 |
| Raw + engineered | Lignin | PLS | 0.485 | 7.54 | 5.22 |
| Raw + engineered | Lignin | SVR | 0.515 | 7.31 | 4.72 |
| Raw + engineered | Lignin | kNN | 0.491 | 7.49 | 4.49 |

# Table S4. Survey of machine-learning applications in bioenergy

*Representative recent studies applying machine learning in bioenergy research, referenced in the Introduction.*

| **Application area** | **Key findings** | **Ref.** |
| --- | --- | --- |
| Gasification modelling | Improved process control and predictive control in conventional and hydrothermal gasification, supporting better decision-making. | [23,35] |
| Pyrolysis and product management | Better prediction of product yields and optimisation of biorefinery output. | [33,52] |
| HHV prediction | Data-driven models predicted higher heating value for biomass classification and energy-conversion optimisation. | [32] |
| Structural composition analysis | ML predicted cellulose, hemicellulose and lignin fractions with R² ≈ 0.88–0.91. | [8,12] |
| Chemical looping / thermochemical optimisation | Reliable forecasts of hydrogen and char yields and optimised operating conditions. | [37,52] |
| Ensemble methods (RF, GB, XGB) | More accurate and robust than traditional ML in several energy applications; application to biomass composition, especially bamboo, remains underdeveloped. | [8,23,24,32,37,38,54–56] |

References:

[72] M. Asif, L. Hakeem, C. Yao, Hira, R. Bibi, M. Bilal, H. Zeb, Machine learning-based prediction of biomass pyrolysis kinetics: integrating mechanistic modeling and compositional features, RSC Advances 16 (2026) 28036–28047. https://doi.org/10.1039/D6RA01011C.

[73] D.R. Roberts, V. Bahn, S. Ciuti, M.S. Boyce, J. Elith, G. Guillera-Arroita, S. Hauenstein, J.J. Lahoz-Monfort, B. Schröder, W. Thuiller, D.I. Warton, B.A. Wintle, F. Hartig, C.F. Dormann, Cross-validation strategies for data with temporal, spatial, hierarchical, or phylogenetic structure, Ecography 40 (2017) 913–929. <https://doi.org/10.1111/ecog.02881>.

[74] Vassilev SV, Baxter D, Andersen LK, Vassileva CG. An overview of the chemical composition of biomass. Fuel 89(5) (2010) 913–933. doi:10.1016/j.fuel.2009.10.022

[75] Vassilev SV, Baxter D, Andersen LK, Vassileva CG. An overview of the organic and inorganic phase composition of biomass. Fuel 94 (2012) 1–33. doi:10.1016/j.fuel.2011.09.030

[76] Sluiter JB, Ruiz RO, Scarlata CJ, Sluiter AD, Templeton DW. Compositional analysis of lignocellulosic feedstocks. 1. Review and description of methods. Journal of Agricultural and Food Chemistry 58(16) (2010) 9043–9053. doi:10.1021/jf1008023

[77] Phyllis2 — Database for the physicochemical composition of (treated) lignocellulosic biomass, micro- and macroalgae. TNO, Netherlands. https://phyllis.nl

[78] Wang X, Yang S, Shen B, Yang J, Xu L. Pyrolysis of biomass pineapple residue and banana pseudo-stem: kinetics, mechanism and valorization of bio-char. Catalysts 12(8) (2022) 840. doi:10.3390/catal12080840

[79] Setiawan A, Zakarya M, Alchalil, Nur TB. Experimental investigation and simulation of slow pyrolysis process of Arabica coffee agroindustry residues in a pilot-scale reactor. Journal of Ecological Engineering 23(8) (2022) 260–269. doi:10.12911/22998993/150693

[80] Sarkar JK, Wang Q. Characterization of pyrolysis products and kinetic analysis of waste jute stick biomass. Processes 8(7) (2020) 837. doi:10.3390/pr8070837

[81] Mohammed IY, Abakr YA, Kazi FK, Yusup S, Alshareef I, Chin SA. Comprehensive characterization of Napier grass as a feedstock for thermochemical conversion. Energies 8(5) (2015) 3403–3417. doi:10.3390/en8053403

[82] Jeguirim M, Dorge S, Loth A, Trouvé G. Devolatilization kinetics of Miscanthus straw from thermogravimetric analysis. International Journal of Green Energy 7(2) (2010) 164–173. doi:10.1080/15435071003673641

[83] Reza MS, Afroze S, Azad AK, Sukri RS, Shams S, Taweekun J, Saghir M, Phusunti N, Abu Bakar MS. Thermochemical characterization of invasive Axonopus compressus grass as a renewable energy source. IOP Conf. Ser.: Materials Science and Engineering 991 (2020) 012074. doi:10.1088/1757-899X/991/1/012074

[84] Ma Z, Chen D, Gu J, Bao B, Zhang Q. Determination of pyrolysis characteristics and kinetics of palm kernel shell using TGA–FTIR and model-free integral methods. Energy Conversion and Management 89 (2015) 251–259. doi:10.1016/j.enconman.2014.09.074

[85] Kumar A, Wang L, Dzenis YA, Jones DD, Hanna MA. Thermogravimetric characterization of corn stover as gasification and pyrolysis feedstock. Biomass and Bioenergy 32(5) (2008) 460–467. doi:10.1016/j.biombioe.2007.11.004

[86] Singh S, Sawarkar AN. Pyrolysis of corn cob: physico-chemical characterization, thermal decomposition behavior and kinetic analysis. Chemical Product and Process Modeling 16(2) (2020) 117–127. doi:10.1515/cppm-2020-0048

[87] Pitchai T, Babu R, Ramanujam S, Kuttalam I. Thermogravimetric and kinetic study of pyrolysis of corn cob biomass. Journal of Renewable Energy and Environment (JREE) 6(4), Serial No. 18 (Autumn 2019) 35–40. doi:10.30501/jree.2019.102816

[88] Onyelucheya CM, Nwabanne JT, Daramola MO, Iwarere SA. Chemical, thermogravimetric and elemental characterization of Nigerian groundnut and cowpea shells for sustainable valorization. IOP Conf. Ser.: Earth Environ. Sci. 1322 (2024) 012003. doi:10.1088/1755-1315/1322/1/012003

[89] Ren X, Guo J, Li S, Chang J. Thermogravimetric analysis–Fourier transform infrared spectroscopy study on the effect of extraction pretreatment on the pyrolysis properties of eucalyptus wood waste. ACS Omega (2020). doi:10.1021/acsomega.0c03271

[90] Orsoletta GD, de Lara Moreira Mandelli MK, da Rocha RDC, Dekker RFH, Cunha MAA. Potential of peanut shells as a biosorbent of Reactive Blue 203 dye in industrial effluent. International Journal of Environmental Science and Technology 22 (2025). doi:10.1007/s13762-024-05847-7

[91] Peng Y, Wu S. The structural and thermal characteristics of wheat straw hemicellulose. Journal of Analytical and Applied Pyrolysis 88(2) (2010) 134–139. doi:10.1016/j.jaap.2010.03.006

[92] Polat S, Apaydın-Varol E, Pütün AE. TGA-FTIR study on the thermal decomposition of tea waste. In: Ozdemir C, Şahinkaya S, Kalıpcı E, Oden MK (eds), Digital Proceeding of the ICOEST'2013, Cappadocia, Nevşehir, Turkey, June 18–21, 2013, pp. 420–428

[93] Slopiecka K, Bartocci P, Fantozzi F. Thermogravimetric analysis and kinetic study of poplar wood pyrolysis. Applied Energy 97 (2012) 491–497. doi:10.1016/j.apenergy.2011.12.056 — your title differed slightly; use whichever you actually read.

[94] Sukarni S, Putra AA, Prasetiyo A, Zakaria Y, Aminullah AY, Permanasari AA, Puspitasari P. Horowitz-Metzger kinetic analysis of coconut shell waste combustion using thermogravimetric technique. AIP Conference Proceedings 3124(1) (2024) 070003. doi:10.1063/5.0228173
